# Loss of the Extracellular Matrix Molecule Tenascin-C Leads to Absence of Reactive Gliosis and Promotes Anti-inflammatory Cytokine Expression in an Autoimmune Glaucoma Mouse Model

**DOI:** 10.3389/fimmu.2020.566279

**Published:** 2020-10-09

**Authors:** Susanne Wiemann, Jacqueline Reinhard, Sabrina Reinehr, Zülal Cibir, Stephanie C. Joachim, Andreas Faissner

**Affiliations:** ^1^Department of Cell Morphology and Molecular Neurobiology, Faculty of Biology and Biotechnology, Ruhr University Bochum, Bochum, Germany; ^2^Experimental Eye Research Institute, University Eye Hospital, Ruhr University Bochum, Bochum, Germany

**Keywords:** autoimmune glaucoma model, cytokines, microglia, optic nerve demyelination, reactive gliosis, tenascin-C

## Abstract

Previous studies demonstrated that retinal damage correlates with a massive remodeling of extracellular matrix (ECM) molecules and reactive gliosis. However, the functional significance of the ECM in retinal neurodegeneration is still unknown. In the present study, we used an intraocular pressure (IOP) independent experimental autoimmune glaucoma (EAG) mouse model to examine the role of the ECM glycoprotein tenascin-C (Tnc). Wild type (WT ONA) and Tnc knockout (KO ONA) mice were immunized with an optic nerve antigen (ONA) homogenate and control groups (CO) obtained sodium chloride (WT CO, KO CO). IOP was measured weekly and electroretinographies were recorded at the end of the study. Ten weeks after immunization, we analyzed retinal ganglion cells (RGCs), glial cells, and the expression of different cytokines in retina and optic nerve tissue in all four groups. IOP and retinal function were comparable in all groups. Although RGC loss was less severe in KO ONA, WT as well as KO mice displayed a significant cell loss after immunization. Compared to KO ONA, less βIII-tubulin^+^ axons, and downregulated oligodendrocyte markers were noted in WT ONA optic nerves. In retina and optic nerve, we found an enhanced GFAP^+^ staining area of astrocytes in immunized WT. A significantly higher number of retinal Iba1^+^ microglia was found in WT ONA, while a lower number of Iba1^+^ cells was observed in KO ONA. Furthermore, an increased expression of the glial markers *Gfap, Iba1, Nos2*, and *Cd68* was detected in retinal and optic nerve tissue of WT ONA, whereas comparable levels were observed in KO ONA. In addition, pro-inflammatory *Tnfa* expression was upregulated in WT ONA, but downregulated in KO ONA. Vice versa, a significantly increased anti-inflammatory *Tgfb1* expression was measured in KO ONA animals. We conclude that Tnc plays an important role in glial and inflammatory response during retinal neurodegeneration. Our results provide evidence that Tnc is involved in glaucomatous damage by regulating retinal glial activation and cytokine release. Thus, this transgenic EAG mouse model for the first time offers the possibility to investigate IOP-independent glaucomatous damage in direct relation to ECM remodeling.

## Introduction

Glaucomatous neurodegeneration is characterized by a progressive loss of retinal ganglion cells (RGCs) and their axons, which form the optic nerve. The molecular mechanisms of RGC degeneration are still not fully understood. In addition to increased intraocular pressure (IOP), immunological processes, glial activation, and remodeling of extracellular matrix (ECM) constituents are associated with glaucoma. In regard to the immune system, studies also indicate an alteration in serum antibodies against various retinal proteins in glaucoma patients with a normal IOP (1–3). The connection between an immune response and glaucoma disease with the characteristic loss of RGCs was already demonstrated in an experimental autoimmune glaucoma (EAG) rat model. Here, glaucomatous damage was induced by immunization with ocular proteins (4, 5). Furthermore, a pathological upregulation of specific ECM components could be demonstrated in this model (6). However, the relationship between a change in ECM components and glaucoma pathogenesis needs to be investigated.

The ECM consists of several molecules, including proteoglycans and glycoproteins and controls cellular key events such as adhesion, differentiation, migration, proliferation as well as survival (7–12). During development of the central nervous system (CNS), tenascin-C (Tnc) is strongly expressed by radial glial cells, immature astrocytes, and oligodendrocyte precursor cells (OPCs). It regulates axonal as well as neurite outgrowth and glial cells differentiation (11, 13–17). In this regard, axonal growth is modulated by specific Tnc isoforms, which contain a different combination of fibronectin type III domains (18). Moreover, Tnc expression is associated with the stem cell niche and regulates stem cell maintenance in addition to differentiation (19).

In the diseased brain, Tnc is highly re-expressed (11). For example, Tnc immunoreactivity is directly linked to amyloid-β plaques in Alzheimer's disease patients (20). A lack of Tnc leads to reduced deposits of amyloid-β plaques and protects from Alzheimer's disease (21). Furthermore, an immunomodulatory impact of Tnc was described by regulating the Th17 cell differentiation and activation (22, 23). Here, Tnc deficiency ameliorates experimental autoimmune encephalomyelitis in mice (24).

In the context of retinal neurodegenerative processes, Tnc also plays an important role (25). During retinal development, Tnc is found in the inner neuroblastic layer at embryonic day 13 (26). In the adult retina, Tnc is highly enriched in the outer and inner plexiform layers and is prominently expressed by amacrine and horizontal cells (27, 28). Additionally, optic nerve astrocytes synthesize Tnc (29, 30). ECM molecules can provide an inhibitory environment for neural regeneration and migration in the retina (30). A dramatic remodeling of ECM constituents was already described after ischemia and glaucomatous damage (25, 31). An upregulation of Tnc has been noted in a glaucoma animal model (32) as well as in open-angle glaucoma patients (33).

Tnc is a key regulator of the immune system and plays an important role during neuroinflammation and glial response (34–36). Moreover, expression of Tnc by astrocytes is regulated by cytokines secreted by microglia (37, 38). Microglia play an important role during neurodegenerative and neuroinflammatory processes (39). Their activation is characterized by an enhanced proliferation, migration, phagocytosis, and increased expression levels of neuroinflammatory molecules (40, 41). The neurotoxic M1-subtype has an amoeboid morphology and releases pro-inflammatory signaling molecules, like tumor necrosis factor-alpha (TNF-α) and inducible nitric oxide synthase (iNOS) (42–44). In contrast, the M2-phenotype is characterized by a morphology with ramified processes and the expression of anti-inflammatory cytokines, such as the transforming growth factor-beta (TGF-β) (45–47).

In this study, we used a Tnc deficient EAG mouse model to further analyze the importance of Tnc during retinal neurodegeneration and neuroinflammatory outcomes. We immunized wild type (WT) and Tnc knockout (KO) mice with an optic nerve antigen (ONA) homogenate and examined retinal and optic nerve damage as well as macro- and microglial activity. Furthermore, we determined the expression pattern of pro- and anti-inflammatory cytokines. The present study was undertaken to address the role of Tnc in glaucomatous damage, retinal glial activation, myelination, and inflammatory cytokine release.

## Materials and Methods

### Animals

Animals were housed under a 12 h light-dark cycle and had free access to chow and water. All procedures were approved by the animal care committee of North Rhine-Westphalia, Germany and performed according to the ARVO statement for the use of animals in ophthalmic and vision research. For the experiments, male and female littermates of 129/Sv WT and *Tnc* KO mice (48) were used at 6 weeks of age.

### Immunization

WT (WT ONA) and KO (KO ONA) mice were immunized intraperitoneally with ONA (1 mg/ml) mixed with incomplete Freund's adjuvants (FA; 50 μl) and 1 μg pertussis toxin (PTx; both Sigma Aldrich, St. Louis, MO, USA) as described (49). To generate the ONA homogenate, fresh bovine eyes were obtained from a local slaughterhouse (Schlachthaus Wuppertal, Germany). As previously described, optic nerves were cut off behind the optic nerve head, cleaned from surrounding tissue and the dura mater was removed. Nerves were pulverized in a cooled mortar and then suspended in phosphate-buffered saline (PBS) (5). A final concentration of 1 mg/ml was set. FA acted as an immunostimulatory and PTx was given to ensure the permeability of the blood retina barrier. Intraperitoneal PTx-application was repeated 2 days after immunization. Booster injections containing half of the initial dose were given intraperitoneally 4 and 8 weeks after initial immunization. The control groups (WT CO; KO CO) were injected with 1 ml sodium chloride (B. Braun Melsungen AG, Melsungen, Germany), FA and PTx. Ten weeks after immunization, retinae, and optic nerves were explanted for immunohistochemistry, quantitative real time PCR (RT-qPCR), and Western blot analyses. For RT-qPCR and Western blot, retinal as well as optic nerve tissue of both eyes from one animal were pooled.

### Intraocular Pressure Measurements

Before IOP measurement, mice were anesthetized with a ketamine/xylazine mixture (120/16 mg/kg). Both eyes were analyzed and 10 readings of each eye were averaged. IOP measurements were performed before immunization in WT and KO mice at 5 weeks of age with a rebound tonometer (TonoLab; Icare; Oy; Finland; *n* = 16/group) as previously described (50, 51). After immunization, IOP was measured weekly in all four groups until the end of the study (*n* = 8/group).

### Electroretinogram Recordings

Scotopic full-field flash electroretinograms (ERG) recordings (HMsERG system, OcuScience, Henderson, NV, USA) were taken 10 weeks after immunization in all groups (*n* = 5/group) as previously described (51). Mice were dark-adapted and anesthetized with a ketamine/xylazine mixture (120/16 mg/kg). Scotopic flash series with flash intensities at 0.1, 0.3, 1.0, 3.0, 10.0, and 25.0 cd/m^2^ were recorded. Electrical potentials were analyzed with the ERGView 4.380R software (OcuScience) using a 150 Hz filter before evaluating a- and b-wave amplitudes.

### Immunohistochemistry and Confocal Laser Scanning Microscopy

Eyes and optic nerves were dissected and fixed in paraformaldehyde (PFA) for 1 day, dehydrated in sucrose (30%) and embedded in Tissue-Tek freezing medium (Thermo Fisher Scientific, Cheshire, UK). Retinal cross-sections and optic nerve longitudinal sections (16 μm) were cut with a cryostat (CM3050 S, Leica) and transferred onto Superfrost plus object slides (Menzel-Glaeser, Braunschweig, Germany). First, slices were blocked with 1% bovine serum albumin (BSA; Sigma-Aldrich), 3% goat serum (Dianova, Hamburg, Germany), and 0.5 % Triton™-X-100 (Sigma-Aldrich) in PBS for 1 hour (h) at room temperature (RT). Afterwards, the primary antibodies were diluted in blocking solution and incubated at RT overnight (Table 1). Sections were washed 3 times in PBS and incubated for 2 h with adequate secondary antibody (Dianova, Hamburg, Germany; Table 1) solution without Triton™-X-100. Cell nuclei were detected with TO-PRO-3 (1:400; Thermo Fisher Scientific). The retinal and optic nerve slices were analyzed with a confocal laser-scanning microscope (LSM 510 META; Zeiss, Göttingen, Germany). Two sections per slide, 4 images per retina (400x magnification), and 3 images per optic nerve (200x magnification) were captured (*n* = 4–5/group). In addition, a 630x magnification was used for colocalization staining in optic nerve sections with antibodies against CC1 (coiled-coil 1) and Olig2 (oligodendrocyte transcription factor 2). Accordingly, 4 images were taken per slide (*n* = 5/group).

**Table 1:** List of primary and secondary antibodies to examine RGCs, macro-, and microglial cell types as well as Tnc in retinae and optic nerves via immunohistochemistry.

**Primary antibody**	**Species, clonality/type**	**Dilution**	**Source (stock no.)/RRID**	**Secondary antibody**	**Species, type**	**Dilution/source**
Brn-3a (flat-mount/ retina)	Goat, polyclonal, IgG	1:300	Santa Cruz (Sc-31984) RRID:AB_2167511	Anti-goat Cy3	Donkey, IgG	1:250/Dianova
CC1 (optic nerve)	Mouse, monoclonal, IgG	1:100	Abcam (Ab16794) RRID:AB_443473	Anti-mouse Cy2	Goat, IgG	1:250/Dianova
GFAP (retina/optic nerve)	Mouse, monoclonal, IgG	1:300	Sigma-Aldrich (G3893) RRID:AB_477010	Anti-mouseCy2	Goat, IgG	1:250/Dianova
Iba1 (flat-mount)	Rabbit, polyclonal, IgG	1:250	WAKO (019-19741) RRID:AB_839504	Anti-rabbit Cy2	Donkey, IgG	1:250/Dianova
MBP (optic nerve)	Rat, monoclonal, IgG	1:250	Bio-Rad (MCA409) RRID:AB_325004	Anti-rat Cy2	Goat, IgG	1:250/Dianova
Olig2 (optic nerve)	Rabbit, polyclonal, IgG	1:400	Merck (AB9610) RRID:AB_570666	Anti-rabbit Cy3	Goat, IgG	1:250/Dianova
Tnc (Kaf12) (retina)	Rabbit, polyclonal, IgG	1:250	(52)	Anti-rabbit Cy3	Goat, IgG	1:250/Dianova
βIII-tubulin (optic nerve)	Mouse, monoclonal, IgG2b	1:300	Sigma-Aldrich (T 8660) RRID:AB_477590	Anti-mouse Cy2	Goat, IgG	1:250/Dianova

*Primary and secondary antibodies, including species, clonality/type, dilution, and source/stock number were shown*.

Laser lines and emission filters were adjusted using the Zeiss ZEN black software. Cropping of the images was done using Coral Paint Shop Pro X8 (Coral Corporation, Ottawa, Canada). Masked evaluation of the staining signal was performed with ImageJ software (ImageJ 1.51w, National Institutes of Health; Bethesda, MD, USA) as previously described (6, 53). Images were converted into gray scales and the background was subtracted. Then, the lower and upper threshold values was determined for each image (Table 2). The percentage of the area fraction was measured using an ImageJ macro as previously described (53). This analysis was performed for immunohistochemical stainings against βIII-tubulin, glial fibrillary acidic protein (GFAP), myelin basic protein (MBP), and Tnc. Cell countings were done for immunopositive Brn3a^+^ cells in retinal cross-sections and for Olig2^+^/CC1^+^ cells in optic nerve slices. Values were transferred to Statistica software and the WT CO group was set to 100% (V13.3; StatSoft (Europe), Hamburg, Germany).

**Table 2:** Adjustments for the ImageJ macro.

**Protein**	**Tissue**	**Background subtraction**	**Lower threshold**	**Upper threshold**
βIII-tubulin	Optic nerve	50	29.45	82.18
GFAP	Retina	20	25.00	76.54
	Optic nerve	50	27.50	78.00
MBP	Optic nerve	50	7.62	62.45
Tnc	Retina	50	6.79	78.68

*Data of background subtractions and upper/lower thresholds were present to determine the immunoreactive area [%]*.

### Quantification of RGCs and Microglia in Retinal Flat-Mounts

Eyes were enucleated and fixed in 4% PFA at 4°C for 1 h. The retinae were dissected from the eye and prepared as flat-mounts (*n* = 9/group). The tissue was fixed again in 4% PFA for 5 min and washed 3 times in PBS. Flat-mounts were blocked in 1% BSA, 3% donkey serum, and 2% Triton™-X-100 in PBS at RT for 1 h. Next, incubation was performed with the RGC specific marker Brn3a [brain-specific homeobox/POU domain protein 3a; (54, 55)] and microglia marker Iba1 [ionized calcium-binding adapter molecule 1; (56)] for 2 days at 4°C. Following PBS washing (3 × 20 min), flat-mounts were incubated with secondary antibodies donkey anti-goat Cy3, donkey anti-rabbit Alexa Fluor 488, and TO-PRO-3 (1:400) in blocking solution without Triton™-X-100 at RT for 2 h. Microscopic images were captured using Axio Zoom.V16 (Zeiss, Göttingen, Germany). Flat-mounts were divided into 16 quadrants (200 × 200 μm) and Brn3a^+^ and Iba1^+^ cells were counted. Groups were compared using two-way ANOVA followed by Tukey's *post-hoc* test. The WT CO group was set to 100%.

### Western Blotting

Retinal tissue (*n* = 5/group) was homogenized in 150 μl and optic nerve tissue (*n* = 5/group) in 100 μl lysis buffer (60 mM n-octyl-β-D-glucopyranoside, 50 mM sodium acetate, 50 mM Tris chloride, pH 8.0 and 2 M urea) containing a protease inhibitor cocktail (Sigma-Aldrich) on ice for 1 h. Prior to lysis, the optic nerve tissue was incubated in liquid nitrogen. Subsequently, all samples were centrifuged at 14.000 × g at 4°C for 30 min and the supernatant was applied to determine the protein concentration. A BCA Protein Assay kit (Pierce, Thermo Fisher Scientific, Rockford, IL, USA) was used for retinal tissue. For optic nerves, the Qubit® Protein Assay kit (Life Technologies GmbH, Darmstadt, Germany) was used according to manufacturer's instructions. 4x SDS buffer was added to each protein sample (20 μg) and denaturized at 94°C for 5 min. After separation via SDS-PAGE (10% gels, respectively, 4–12% polyacrylamide gradient gels), proteins were transferred to a polyvinylidene difluoride (PVDF) membrane (Roth, Karlsruhe, Germany) by Western blotting (1–2 h and 75 mA). Membranes were blocked (5% w/v milk powder in TRIS-buffered saline (TBS) with 0.05% Tween®20; TBST) at RT for 1 h and incubated with the primary antibody (Table 3) in blocking solution at 4°C overnight. On the next day, membranes were washed with TBST and incubated with horseradish peroxidase (HRP) coupled secondary antibodies (Table 3) in blocking solution at RT for 2 h. Excess antibody was washed off with TBST. ECL Substrate (Bio-Rad Laboratories GmbH, München, Germany) was applied to develop the membrane (mixed 1:1 for 5 min). Finally, protein immunoreactivity was detected with a MicroChemi Chemiluminescence Reader (Biostep, Burkhardtsdorf, Germany). Band intensity was analyzed using ImageJ software and normalized to a corresponding reference protein (actin/vinculin). The normalized values of the Western blot results were given in arbitrary units (a.u.).

**Table 3:** List of primary and secondary antibodies for western blotting.

**Primary antibody**	**Species, clonality, type**	**Dilution**	**Source (stock no.)/RRID**	**Secondary antibody, species, type**	**Dilution**	**Source**	**kDa**
Actin	Mouse, monoclonal, IgG	1:5,000	BD Bioscience (612657) RRID:AB_399901	Anti-mouse, IgG + IgM HRP	1:5,000	Dianova	42
GFAP	Rabbit, polyclonal, IgG	1:10,000	Dako (Z0334) RRID:AB_10013382	Anti-rabbit, IgG HRP	1:10,000	Dianova	50
MBP	Rat, monoclonal, IgG	1:1,000	Bio-Rad (MCA409) RRID:AB_325004	Anti-rat, IgG HRP	1:5,000	Dianova	20
Olig2	Rabbit, polyclonal, IgG	1:500	Merck (AB9610) RRID:AB_570666	Anti-rabbit, IgG HRP	1:5,000	Dianova	32 and 50
Tnc (Kaf12)	Rabbit, polyclonal, IgG	1:5,000	(52)	Anti-rabbit, IgG HRP	1:5,000	Dianova	~ 250 and > 250
Vinculin	Mouse, monoclonal, IgG1	1:200	Sigma-Aldrich (V 9131) RRID:AB_477629	Anti-mouse, IgG + IgM HRP	1:5,000	Dianova	116

*Primary and secondary antibodies, including species, clonality, type, dilution, source, and stock number are listed. Relative quantification of band intensity was done against the housekeeping proteins actin or vinculin. HRP, horseradish peroxidase; kDa, Kilodalton*.

### RNA Isolation, cDNA Synthesis, and RT-qPCR

Retinal tissue was homogenized by trituration using a pipette (*n* = 5/group). The RNA isolation of the retina was carried out according to the manufacturer's introduction using the Gene Elute Mammalian Total RNA Miniprep Kit (Sigma-Aldrich, St. Louis, MO, USA). For total RNA isolation of optic nerve tissue, the ReliaPrepTM RNA Tissue Miniprep System (Promega, Madison, WI, USA) was taken. Prior to isolation, optic nerve tissue was incubated in liquid nitrogen and then homogenized with a pestle (*n* = 5/group). An additional DNase digestion at RT for 15 min ensured that no genomic DNA contaminated RNA was obtained. The concentration and purity of the isolated RNA was determined photometrically using the BioSpectrometer® (Eppendorf, Hamburg, Germany). One microgram RNA and random hexamer primers were applied for reverse transcription using a cDNA synthesis kit (Thermo Fisher Scientific, Waltham, MA, USA). RT-qPCR experiments were done with SYBR Green I in a Light Cycler 96® (Roche Applied Science, Mannheim, Germany). For each primer pair (Table 4), efficiencies were determined by a dilution series of 5, 25, and 125 ng cDNA. Expression in retina and optic nerve tissue was normalized against the housekeeping genes β*-actin* (*Actb*) and *18S ribosomal RNA (Rn18S)*, respectively.

**Table 4:** List of primer pairs used for mRNA analyses by RT-qPCR.

**Gene**	**Primer sequence**	**Amplicon size (bp)**	**Primer efficiency, retina/optic nerve**	**GenBank accession number**
*Rn18S*_for	GCAATTATTCCCCATGAACG	68	–/1	NR_003278.3
*Rn18S*_rev	GGGACTTAATCAACGCAAGC			
*Actb*_for	CTAAGGCCAACCGTGAAAAG	104	0.84/–	NM_007393.3
*Actb*_rev	ACCAGAGGCATACAGGGACA			
*Cd68*_for	GGACCCACAACTGTCACTCA	60	0.89/1	NM_001291058.1
*Cd68*_rev	AATTGTGGCATTCCCATGAC			
*Gfap*_for	ACAGACTTTCTCCAACCTCCAG	63	0.84/1	NM_001131020
*Gfap*_rev	CCTTCTGACACGGATTTGGT			
*Iba1*_for	GGATTTGCAGGGAGGAAAAG	92	0.80/0.91	NM_019467.3
*Iba1*_rev	TGGGATCATCGAGGAATTG			
*Nos2*_for	CTTTGCCACGGACGAGAC	66	1/0.70	NM_010927.3
*Nos2*_rev	TCATTGTACTCTGAGGGCTGAC			
*Tgfb1*_for	TGGAGCAACATGTGGAACTC	73	1/0.91	NM_011577.2
*Tgfb1*_rev	GTCAGCAGCCGGTTACCA			
*Tnfa*_for	TCTTCTCATTCCTGCTTGTGG	101	1/0.97	NM_013693.3/NM_001278601.1
*Tnfa*_rev	GAGGCCATTTGGGAACTTCT			

*For evaluation of mRNA-levels, Actb, and Rn18S served as housekeeping genes. Primer sequence, predicted amplicon size, primer efficiency for retinae and optic nerves and GenBank accession number are listed. Bp, base pairs; for, forward; rev, reverse*.

### Statistical Analysis

Immunohistological, Western blot, IOP, and ERG data of control WT (WT CO) and KO (KO CO) as well as ONA-immunized WT (WT ONA) and KO (KO ONA) were analyzed by two-way ANOVA followed by Tukey's *post-hoc* test using Statistica software (V13.3; StatSoft (Europe), Hamburg, Germany). Results of IOP measurements were presented as mean ± standard error mean (SEM) ± standard deviation (SD). ERG recordings, immunohistochemical, and Western blot data were shown as mean ± SEM. Analyses of Tnc protein levels in WT mice via immunohistochemistry and Western blot were analyzed via Student *t*-test and presented as mean ± SEM. For RT-qPCR results, groups were compared using the pairwise fixed reallocation and randomization test (REST software) and were presented as median ± quartile ± minimum/maximum (57).

## Results

### No Changes in IOP and Retinal Functionality in the EAG Mouse Model

**Figure 1:**
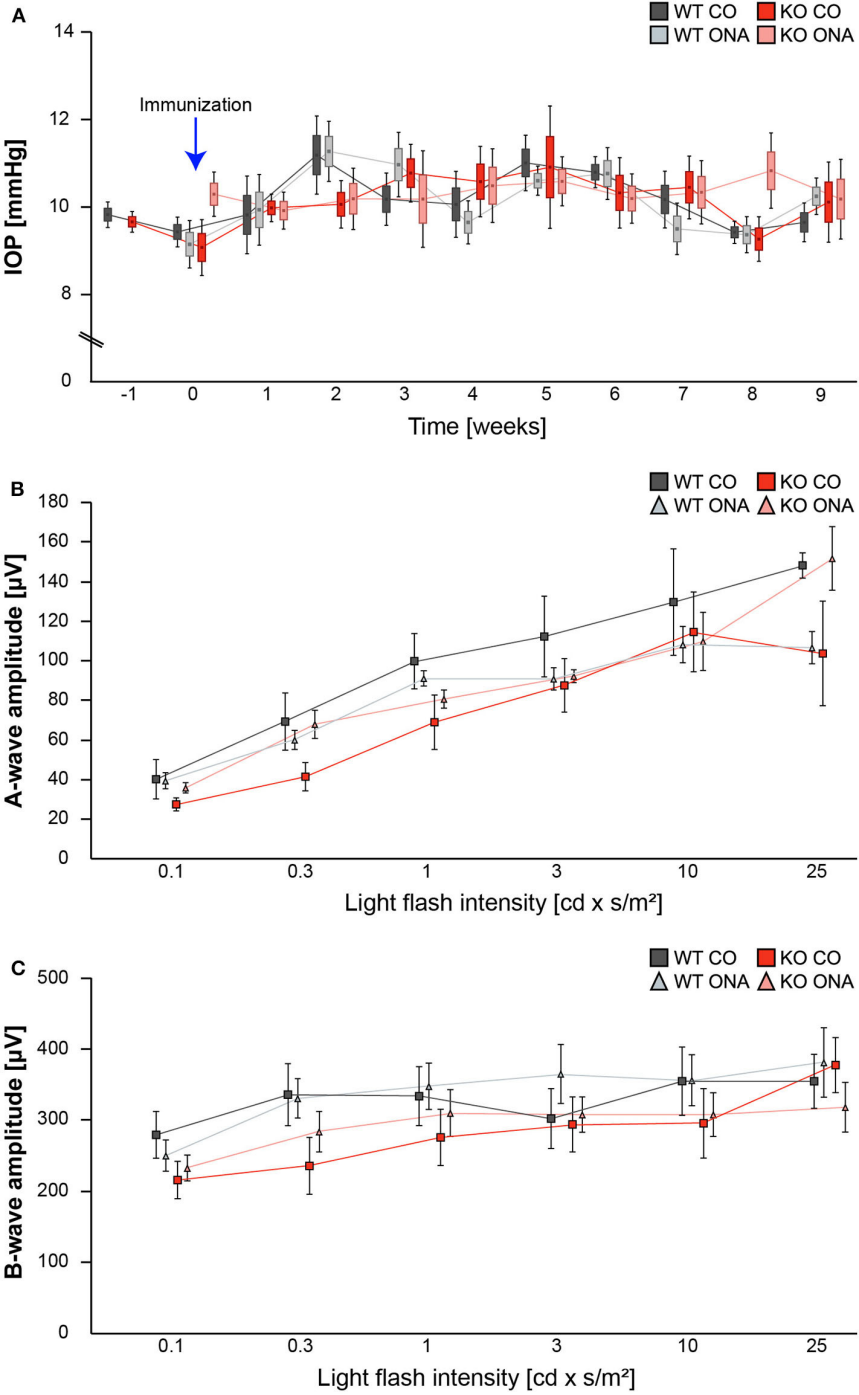
IOP and ERG recordings were not altered after immunization of WT and KO mice. **(A)** IOP measurements were performed before immunization in 5 weeks old WT and KO mice (−1; *n* = 16/group). Then, IOP was determined weekly in immunized and control WT and KO until the end of the study (*n* = 8/group). No significant changes could be detected between all groups. **(B,C)** ERG recordings 10 weeks after initial immunization in control and immunized WT and KO mice. No changes in a-wave **(B)** and b-wave **(C)** amplitudes could be detected in control and immunized WT and KO mice (*n* = 5/group). Data were analyzed using two-way ANOVA followed by Tukey's *post-hoc* test and present as mean ± SEM ± SD in **(A)** and mean ± SEM in **(B,C)**. cd, candela; IOP, intraocular pressure; μV, micro volt; m, minutes; s, seconds.

IOP measurements were performed before immunization in 5-week-old WT (WT CO) and KO (KO CO; Figure 1A). After immunization, IOP was measured weekly in control and immunized WT (WT ONA) and KO (KO ONA) animals until the end of the study. At 5 weeks of age (−1), we observed no significant differences in the IOP of WT CO (9.8 ± 0.2 mmHg) and KO CO (9.7 ± 0.1 mmHg; *p* = 1.0). Furthermore, no changes in the IOP were found in control and immunized groups throughout the study (Supplementary Table 1).

To determine possible retinal function deficits, induced by ONA-immunization, we performed ERG recordings of control and immunized WT and KO mice. Under scotopic conditions, a-wave responses arise from rod-photoreceptors, while b-waves represent the rod bipolar and Müllerglia cell response. In all four conditions no significant differences were observed between control and immunized WT and KO animals (Figures 1B,C; Supplementary Table 2). Therefore, we concluded that photoreceptor and bipolar cell function was not affected in this EAG mouse model.

### Significant RGC Loss Following Immunization

**Figure 2:**
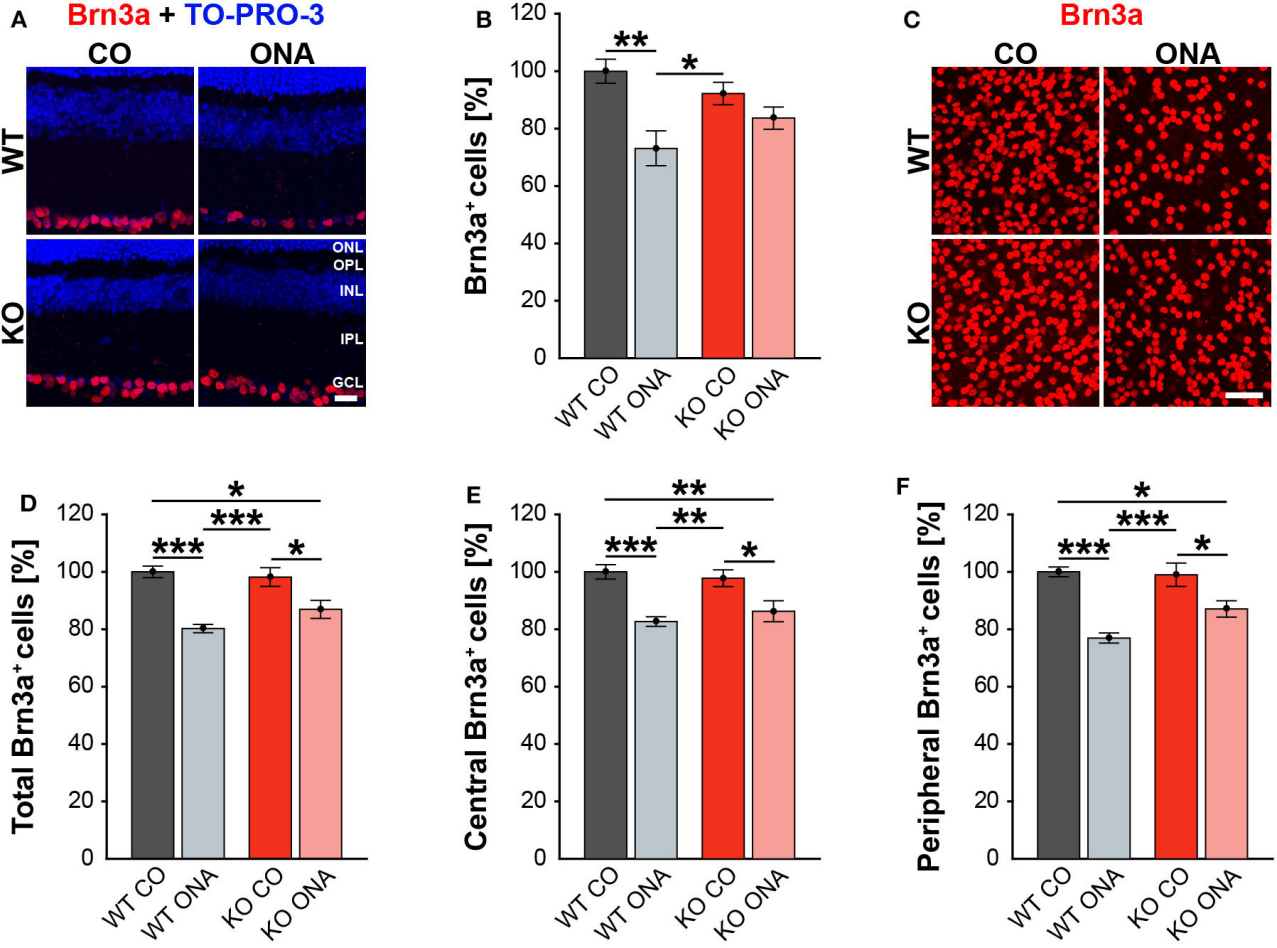
Less RGC loss in immunized KO animals. **(A)** Retinal cross-sections from WT CO, WT ONA, KO CO, and KO ONA mice were stained with an antibody against Brn3a (red) and nuclei were detected with TO-PRO-3 (blue). **(B)** A decline of RGC numbers was detected in WT ONA compared to the control groups (*n* = 5/group). **(C)** Representative pictures of Brn3a^+^ cells in retinal flat-mounts. **(D–F)** Quantification of the total RGC number as well as in central and peripheral parts (*n* = 9/group). A significant loss of RGCs was detected in immunized WT and KO in comparison to the control groups. It was also shown that the RGC number in KO ONA RGCs were significantly decreased compared to WT CO. Furthermore, WT ONA RGC numbers were significantly reduced compared to KO CO. Data were analyzed using two-way ANOVA followed by Tukey's *post-hoc* test and values were shown as mean ± SEM. *p < 0.05; **p < 0.01; ***p < 0.001. Scale bar = 20 μm in **(A)** and 50 μm in **(C)**. ONL, outer nuclear layer; OPL, outer plexiform layer; INL, inner nuclear layer; IPL, inner plexiform layer; GCL, ganglion cell layer.

Previous studies of an EAG rat model showed a significant reduction of RGC numbers 4 weeks post immunization with ONA (4, 58). Additionally, an upregulation of Tnc was found before significant RGC loss occurred (6). In our EAG mouse model, we detected unaltered Tnc protein levels 10 weeks after immunization (Supplementary Table 3, Supplementary Figure 1). Based on these findings, immunohistochemical stainings of RGCs were performed with an antibody against Brn3a, which specifically detects RGCs (Figure 2, Supplementary Table 4). The evaluation of RGCs in retinal cross-sections showed a significant reduction in the percentage of Brn3a^+^ cells in WT ONA compared to WT CO as well as to KO CO (WT ONA: 73.1 ± 6.1% Brn3a^+^ cells vs. WT CO: 100.0 ± 4.2% Brn3a^+^ cells; *p* = 0.004 and KO CO: 92.2 ± 3.9% Brn3a^+^ cells, *p* = 0.04, Figures 2A,B). Interestingly, no significant differences were detected between control and immunized KO in horizontal cross-sections (KO CO: 92.2 ± 3.9% Brn3a^+^ cells vs. KO ONA: 83.7 ± 8.7% Brn3a^+^ cells, *p* = 0.57, Figures 2A,B).

To further characterize the RGC population, we counted Brn3a^+^ cells in retinal flat-mounts (Figure 2C). We determined the total amount (Figure 2D) as well as the number of Brn3a^+^ cells within the central (Figure 2E) and peripheral (Figure 2F) area of the retina. A significant reduction in the total number was observed in immunized WT compared to both control genotypes (WT ONA: 80.3 ± 1.5% Brn3a^+^ cells vs. WT CO: 100.0 ± 2.0% Brn3a^+^ cells, *p* < 0.001, KO CO: 98.2 ± 3.3% Brn3a^+^ cells, *p* < 0.001). Also, a significant loss of RGCs was detected in KO ONA mice (86.9 ± 3.1% Brn3a^+^ cells) compared to KO CO (*p* = 0.02) and WT CO (*p* = 0.01). A comparable percentage of Brn3a^+^ cells was also observed in the central retina. So, immunized WT and KO animals showed a significant decline of RGCs compared to the corresponding control groups (WT ONA: 82.7 ± 1.7% Brn3a^+^ cells vs. WT CO: 100.0 ± 2.5% Brn3a^+^ cells, *p* < 0.001 and KO ONA: 86.3 ± 3.7% Brn3a^+^ cells vs. KO CO: 97.8 ± 2.9% Brn3a^+^ cells, *p* = 0.03). No significant differences were found between both immunized genotypes (*p* = 0.80). Furthermore, a decrease in the RGC density was verified in the peripheral area. Retinae of the WT ONA group (77.0 ± 1.8% Brn3a^+^ cells) displayed a loss of about 25% RGCs compared to WT CO (100.0 ± 1.7% Brn3a^+^ cells, *p* < 0.001). A significant reduction was also found in the comparison of KO CO and KO ONA (KO CO: 99.0 ± 4.1% Brn3a^+^ cells vs. KO ONA: 87.1 ± 2.8% Brn3a^+^ cells, *p* = 0.02). However, KO ONA showed a decrease of about 15 % in the peripheral part compared to WT CO group (*p* = 0.01).

Although not statistically significant, we found a trend to a weaker RGC damage in immunized KO compared to WT ONA.

### Optic Nerve Degeneration Post ONA-Immunization in WT Mice

**Figure 3:**
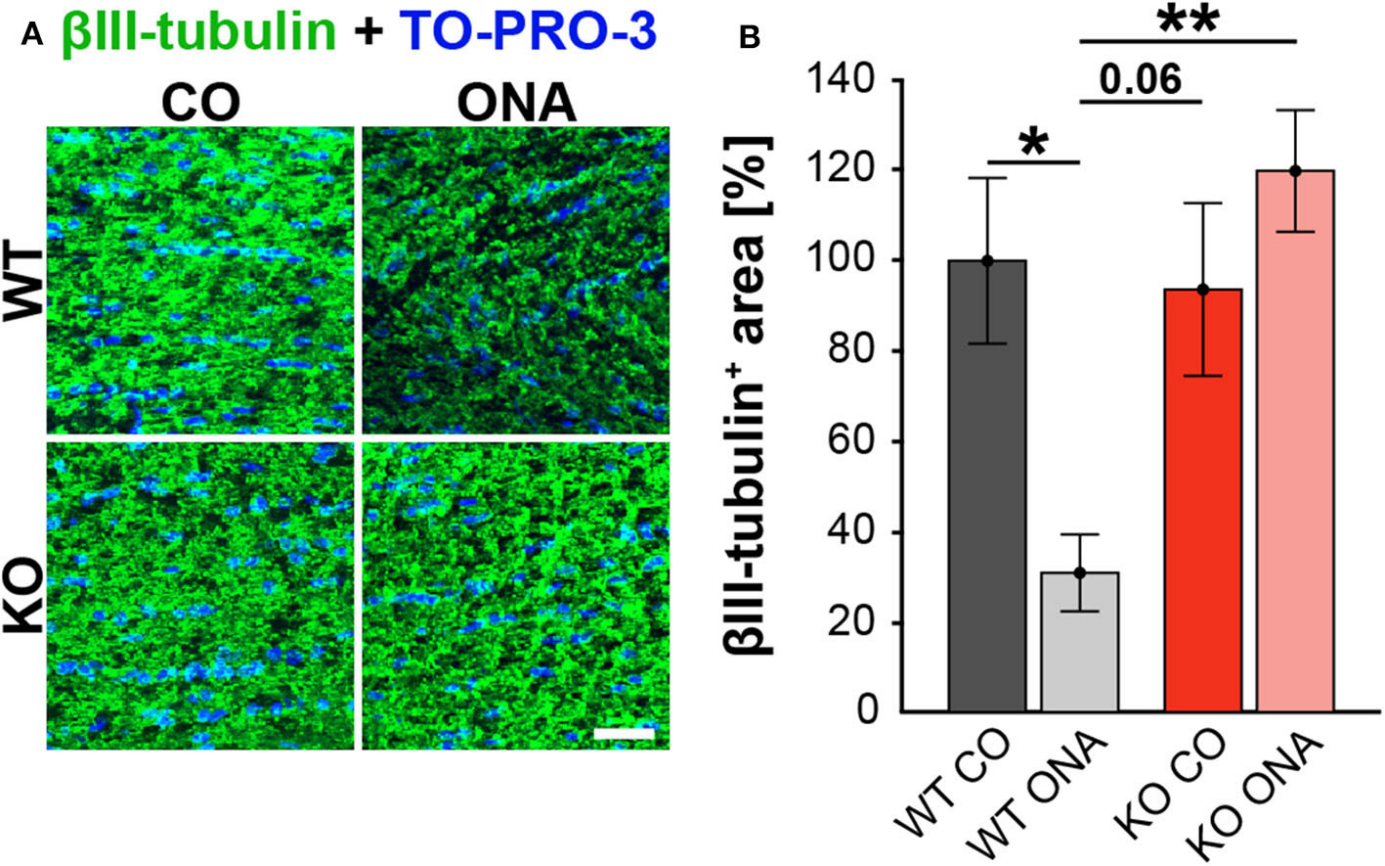
No optic nerve degeneration in KO post immunization. **(A)** Optic nerve slices were stained with βIII-tubulin (green) and cell nuclei were marked with TO-PRO-3 (blue). **(B)** WT ONA mice showed a significantly decreased βIII-tubulin^+^ area compared to WT CO as well as to KO ONA group. Data were analyzed using two-way ANOVA followed by Tukey's *post-hoc* test and presented as mean ± SEM. *p < 0.05; **p < 0.01. *n* = 4/group. Scale bar = 20 μm.

To analyze a possible degeneration of RGC axons, immunoreactivity of βIII-tubulin was examined in optic nerve longitudinal sections of control and immunized WT and KO animals (Figure 3A). The immunopositive βIII-tubulin area was significantly reduced in immunized WT (30.85 ± 8.55% βIII-tubulin^+^ area) compared to WT CO (100.00 ± 18.35% βIII-tubulin^+^ area, *p* = 0.04, Figure 3B). Also, the βIII-tubulin^+^ area was decreased in WT ONA compared to both KO conditions (KO CO: 93.66 ± 19.18% βIII-tubulin^+^ area, *p* = 0.06 and KO ONA: 119.93 ± 13.48% βIII-tubulin^+^ area, *p* < 0.01).

### Extenuated Macroglial Reactivity After Immunization in KO Mice

**Figure 4:**
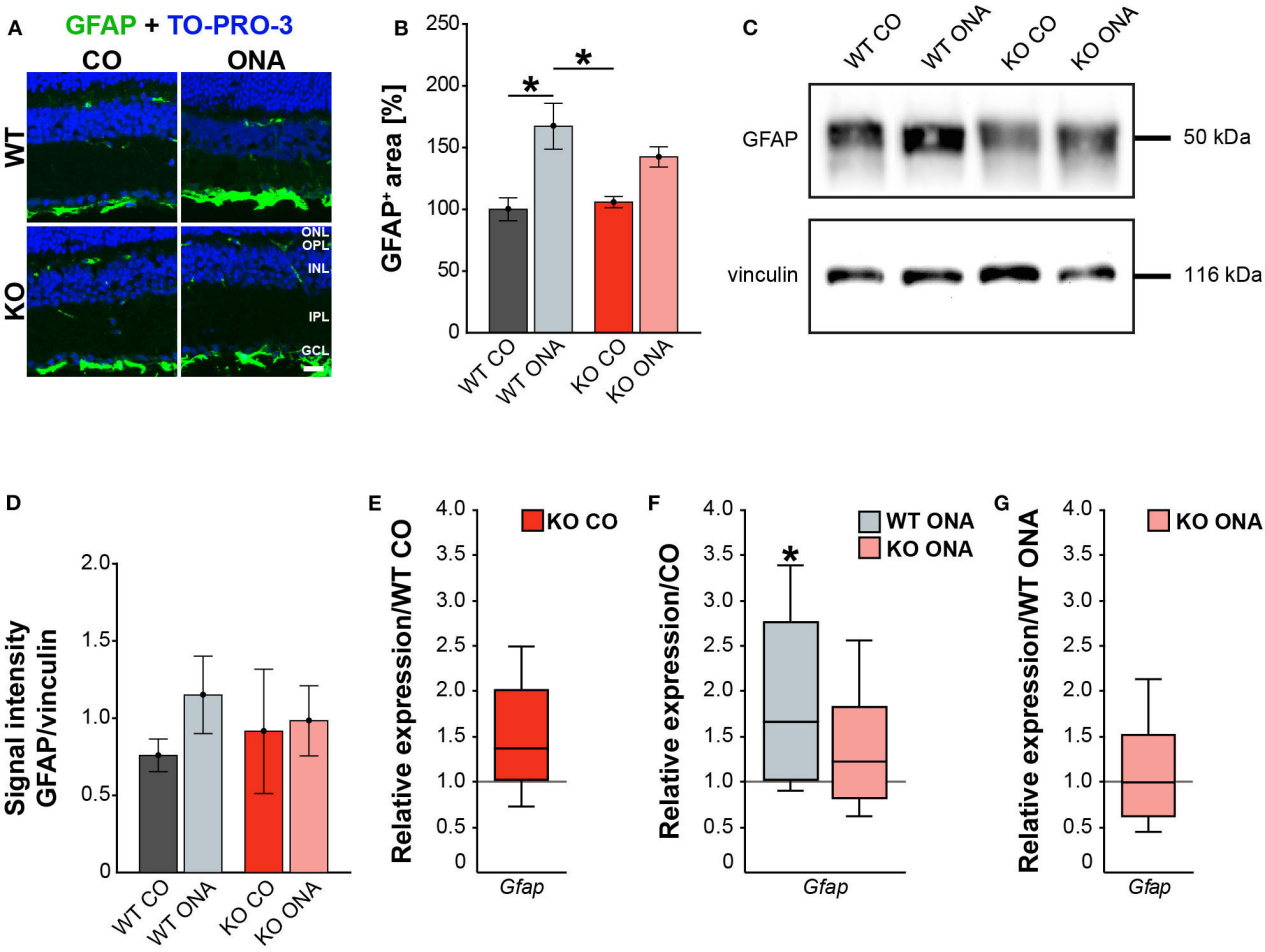
Reduced astrogliosis after immunization in KO mice. **(A)** Images of GFAP stained macroglia cells in retinal cross-section from control and immunized WT and KO animals. Immunohistochemistry revealed a prominent signal for GFAP^+^ cells (green) in the GCL. Cell nuclei were detected with TO-PRO-3 (blue). **(B)** GFAP^+^ area was significantly increased in WT ONA compared to WT CO. No changes of the GFAP signal area were found in KO ONA compared to the control groups. **(C)** Western blot analyses of GFAP protein in retinal tissue. **(D)** Quantification revealed slightly more GFAP in WT ONA, whereas a comparable level was observed in KO ONA. **(E)** No differences of *Gfap* expression were noted in KO CO compared to WT CO. **(F)** A significant upregulation of *Gfap* mRNA expression was seen in WT ONA in comparison to WT CO. **(G)** WT ONA and KO ONA animals showed similar *Gfap* levels. Data were analyzed using two-way ANOVA followed by Tukey's *post-hoc* test and present as mean ± SEM in **(B,D)**. For RT-qPCR results, groups were compared using the pairwise fixed reallocation and randomization test and were shown as median ± quartile ± minimum/maximum in **(E–G)**. *p < 0.05. *n* = 5/group. Scale bar = 20 μm. ONL, outer nuclear layer; OPL, outer plexiform layer; INL, inner nuclear layer; IPL, inner plexiform layer; GCL, ganglion cell layer.

Our results showed a decrease in the RGC number 10 weeks after immunization in WT and KO animals. Next, we investigated, if this glaucomatous neurodegeneration is associated with an altered macroglial response. Therefore, we analyzed the immunoreactivity of GFAP^+^ astrocytes in retinal cross-sections. GFAP stained astrocytes were mainly localized in the ganglion cell layer (GCL; Figure 4A). The GFAP^+^ area was increased in WT ONA (167.22 ± 18.61% GFAP^+^ area) compared to the control groups (WT CO: 100.00 ± 9.28% GFAP^+^ area, *p* = 0.01 and KO CO: 105.81 ± 4.54% GFAP^+^ area, *p* = 0.02, Figure 4B). Interestingly, no changes in the GFAP signal area were found in KO ONA (142.49 ± 8.19% GFAP^+^ area) compared to KO CO (*p* = 0.18) as well as to WT CO (*p* = 0.11). The statistical comparison of both immunized genotypes showed no significant differences (*p* = 0.46).

Then, we evaluated GFAP protein levels via Western blot. For GFAP, a prominent band was detected at 50 kDa (Figure 4C). Relative quantification verified a slight, but not statistically significant, increase in the GFAP protein concentration in WT after immunization (WT ONA: 1.15 ± 0.25 a.u. vs. WT CO: 0.76 ± 0.11 a.u., *p* = 0.73 and KO CO: 0.91 ± 0.40 a.u., *p* = 0.92, Figure 4D). No changes were observed in the GFAP level of control and immunized KO animals (KO ONA: 0.98 ± 0.23 a.u., *p* = 0.99).

We also analyzed the mRNA expression of *Gfap* in retinae via RT-qPCR (Figures 4E–G, Supplementary Table 5). Analysis revealed comparable levels of *Gfap* in KO CO and WT CO (1.4-fold, *p* = 0.11, Figure 4E). A significant increase of *Gfap* mRNA expression levels was observed in WT ONA (WT CO vs. WT ONA: 1.7-fold, *p* = 0.04, Figure 4F), whereas no differences could be detected in KO ONA (KO CO vs. KO ONA: 1.2-fold, *p* = 0.40, Figure 4F). The expression was comparable in both immunized genotypes (1.0-fold, *p* = 0.99, Figure 4G).

**Figure 5:**
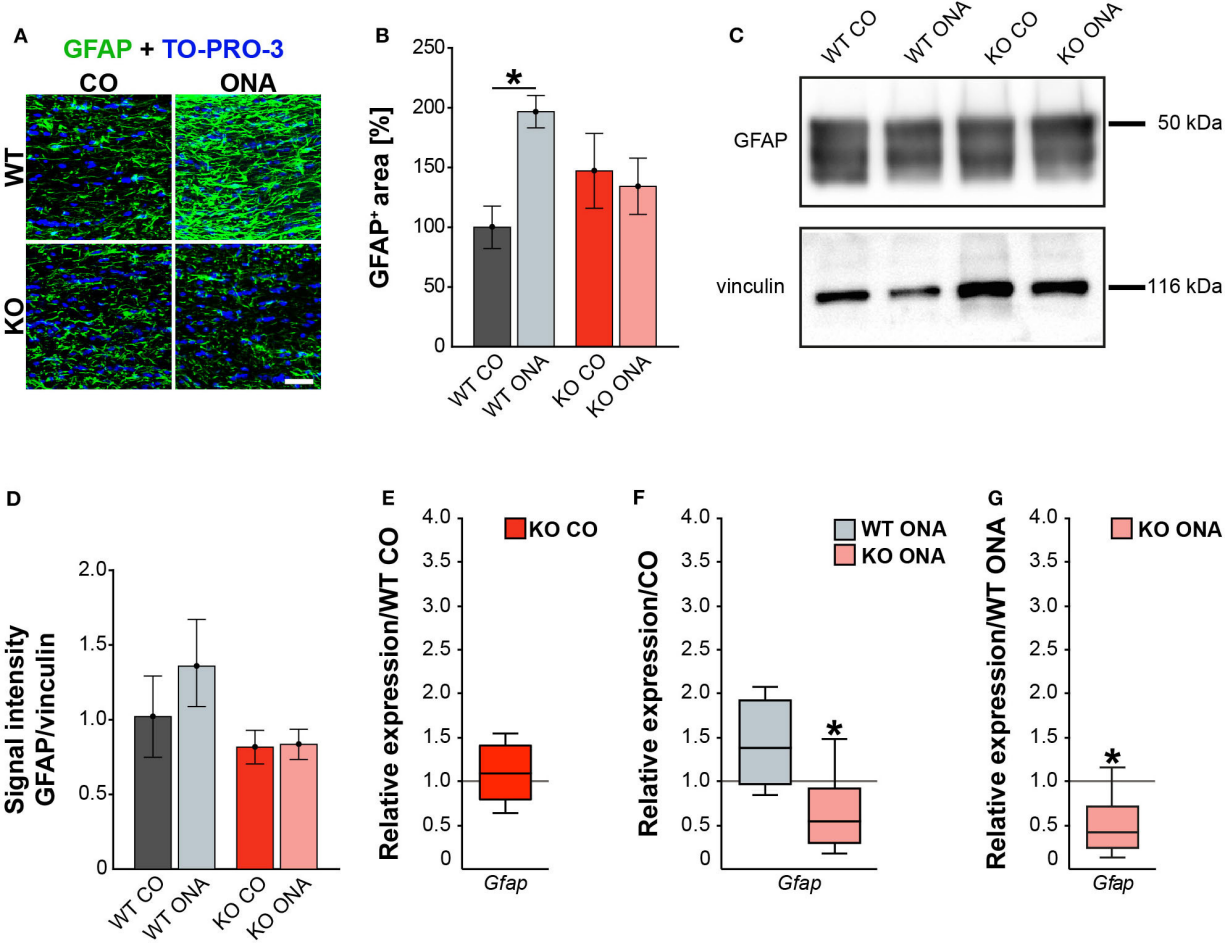
Diminished macroglial response post ONA-immunization in optic nerve tissue of KO animals. **(A)** Representative pictures of optic nerve sections of control and immunized WT and KO mice stained against GFAP (green). Nuclear staining was done with TO-PRO-3 (blue). **(B)** WT ONA animals showed a larger GFAP^+^ staining area then WT CO. No differences between KO mice could be detected. **(C)** Western blot analyses of relative GFAP protein levels in optic nerve tissue. **(D)** Protein quantification revealed slightly enhanced band intensity in WT ONA, whereas KO ONA exhibit no increased GFAP level. **(E)** No differences of *Gfap* expression were noted in KO CO compared to WT CO. **(F)** A slight upregulation of *Gfap* mRNA expression was seen in WT ONA in comparison to WT CO, but in KO ONA decreased expression was verified compared to KO CO. **(G)** A downregulation of *Gfap* in KO ONA in comparison to WT ONA was noted. Data were analyzed using two-way ANOVA followed by Tukey's *post-hoc* test and were shown as mean ± SEM in **(B,D)**. For RT-qPCR, groups were compared using the pairwise fixed reallocation and randomization test and were shown as median ± quartile ± minimum/maximum in **(E–G)**. *p < 0.05. *n* = 5/group. Scale bar = 20 μm.

For GFAP, a thread-like staining pattern was observed in optic nerve slices (Figure 5A). The evaluation of the GFAP immunoreactivity in optic nerve sections also showed no increased macroglial area in KO post immunization (KO ONA: 134.30 ± 23.57% GFAP^+^ area vs. KO CO: 147.18 ± 31.27% GFAP^+^ area, *p* = 0.98 and WT CO: 100.00 ± 17.72% GFAP^+^ area, *p* = 0.70, Figure 5B). Moreover, a nearly doubled GFAP intensity was observed in WT ONA (196.70 ± 13.60% GFAP^+^ area) compared to the corresponding control group (*p* = 0.04).

Furthermore, protein levels of GFAP via Western blot analyses were comparable between all four groups (Figure 5C). However, the band intensity in WT ONA group was tendentially increased compared to the control group (WT ONA: 1.36 ± 0.29 a.u. vs. WT CO: 1.02 ± 0.27 a.u., *p* = 0.68, Figure 5D). Equal protein levels were found between control and KO ONA animals (KO CO: 0.82 ± 0.11 a.u. vs. KO ONA: 0.84 ± 0.10 a.u., *p* = 0.99).

Finally, the RT-qPCR results of the optic nerve tissue showed no changes in *Gfap* expression between both control groups (WT CO vs. KO CO: 1.1-fold, *p* = 0.54, Figure 5E, Supplementary Table 5). In line with the immunohistochemical results, we found a slightly enhanced mRNA level in WT ONA (WT CO vs. WT ONA: 1.4-fold, *p* = 0.07, Figure 5F), whereas the KO ONA animals exhibited a reduction of *Gfap* expression (KO CO vs. KO ONA: 0.5-fold, *p* < 0.05). Interestingly, in a direct comparison of the two immunized groups, *Gfap* expression was significantly reduced in KO animals (WT ONA vs. KO ONA: 0.4-fold, *p* = 0.02, Figure 5G).

In summary, we concluded that Tnc deficiency resulted in a diminished macroglial reaction during retinal and optic nerve degeneration in the EAG mouse model.

### Decreased Demyelination After ONA-Immunization in KO Mice

**Figure 6:**
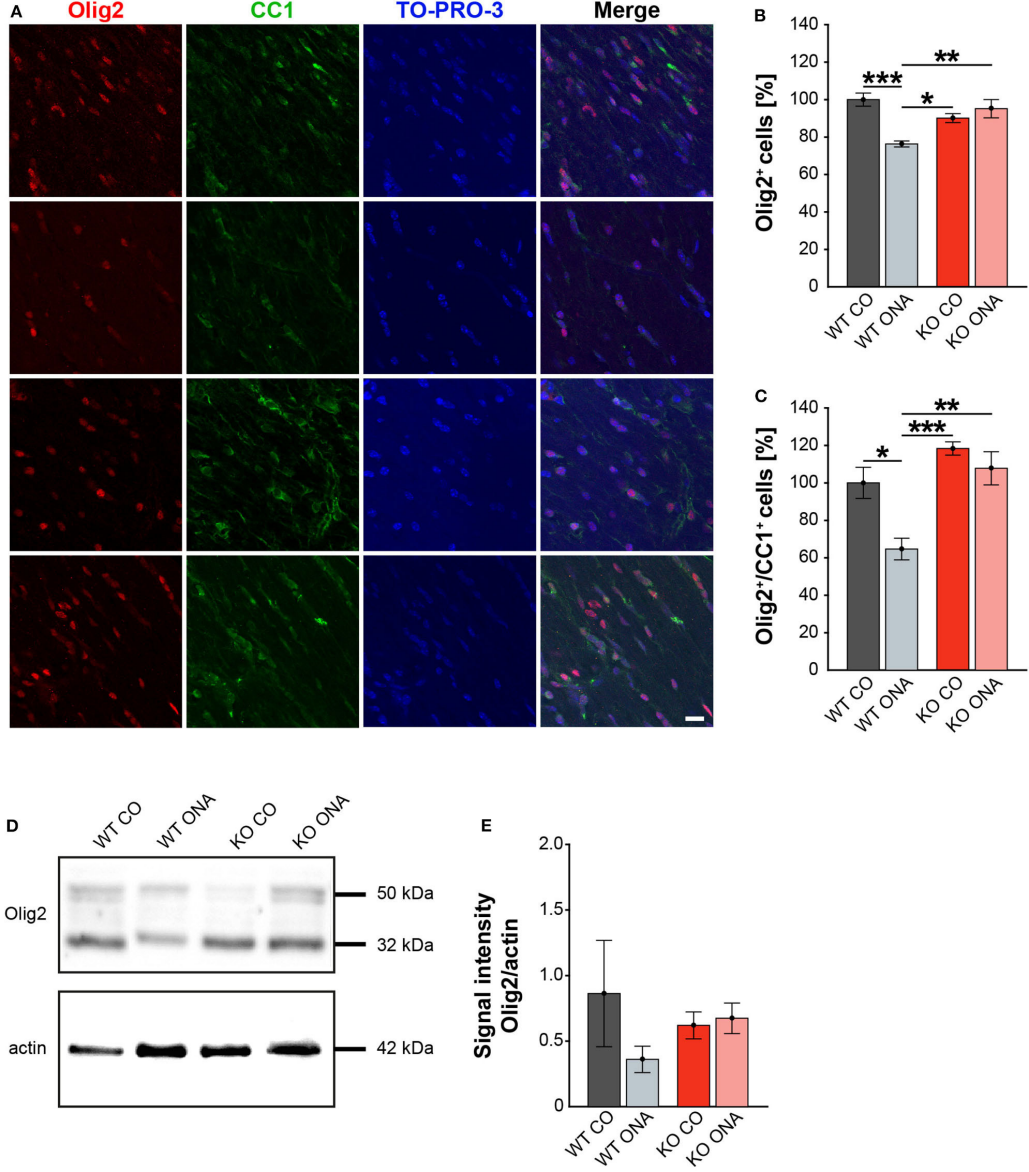
No demyelination after immunization in KO mice. **(A)** Olig2 (red) and CC1 (green) staining of optic nerve sections. Cell nuclei were labeled with TO-PRO-3 (blue). **(B)** Quantification of Olig2^+^ cells revealed a significant decrease of oligodendroglia in WT ONA compared to WT CO and KO CO. Interestingly, the statistical comparison of both immunized groups showed a significant loss of Olig2^+^ cells in WT compared to KO mice. **(C)** WT ONA nerves displayed a significantly decrease of mature oligodendrocytes in comparison to both control groups. A significantly higher amount of double positive oligodendrocytes was observed in KO ONA compared to WT ONA. **(D)** An exemplary Western blot of Olig2. **(E)** Relative protein quantification revealed a slightly decreased band intensity of the Olig2 protein in WT ONA, whereas KO ONA nerves exhibited no reduction of the Olig2 protein level. Data were analyzed using two-way ANOVA followed by Tukey's *post-hoc* test and values were shown as mean ± SEM. *p < 0.05; **p < 0.01, ***p < 0.001. *n* = 5/group. Scale bar = 20 μm.

Our study demonstrates RGC degeneration in WT and KO animals after immunization. Furthermore, we noted that Tnc deficiency resulted in a diminished macroglial response. Finally, we analyzed the impact of ONA-immunization on oligodendroglia in optic nerve tissue. The oligodendrocytes appear in two different populations, as immature OPCs and as myelinating, mature oligodendrocytes. To analyze both oligodendrocyte populations separately, an immunohistochemical colocalization staining was performed using the markers Olig2 and CC1. Olig2 is expressed by oligodendrocytes of all stages (59). In contrast, CC1 is only expressed by mature oligodendrocytes (60). Colocalization identified double positive cells as mature and single Olig2^+^ cells as immature oligodendrocytes. Immunohistochemical stainings revealed fewer Olig2^+^ cells in the WT ONA group compared to the other groups. Interestingly, there were more Olig2^+^ cells in KO ONA than in WT ONA (Figure 6A). 76.3 ± 1.6% Olig2^+^ cells were found in WT ONA, which indicates a significant oligodendrocyte loss over 25% compared to control WT (100.0 ± 3.5% Olig2^+^ cells, *p* < 0.001, Figure 6B). The number of Olig2^+^ cells was also significantly decreased in WT ONA compared to KO CO (*p* = 0.04). No differences were observed between both Tnc deficient groups (KO CO: 90.2 ± 2.4% Olig2^+^ cells vs. KO ONA: 95.2 ± 4.9% Olig2^+^ cells, *p* = 0.71). Most interestingly, we verified significant differences between both immunized groups (*p* < 0.01). The number of double-positive (Olig2^+^/CC1^+^) mature oligodendrocytes was clearly reduced in WT ONA compared to all other groups (Figure 6A). The statistical evaluation demonstrated only 64.7 ± 5.8% Olig2^+^/CC1^+^ cells in WT ONA, whereas KO ONA exhibited 107.8 ± 8.9% Olig2^+^/CC1^+^ cells in optic nerve slices (*p* = 0.002, Figure 6C). Also, immunized WT showed a significant loss of mature oligodendrocytes compared to WT CO (100.0 ± 8.3% Olig2^+^/CC1^+^ cells, *p* = 0.01) and KO CO (118.4 ± 3.5% Olig2^+^/CC1^+^ cells, *p* < 0.001).

To consolidate the immunohistochemistry results, we analyzed the Olig2 protein level in optic nerves by Western blot analyses (Figure 6D). For Olig2, we observed two bands at 32 and 50 kDa. A slight but not significantly decrease of the band intensity was found in WT ONA (0.36 ± 0.10 a.u.) compared to the corresponding control group (0.86 ± 0.41 a.u., *p* = 0.41, Figure 6E). Equal Olig2 protein levels were observed in KO CO (0.62 ± 0.10 a.u.) and KO ONA (0.67 ± 0.12 a.u., *p* = 0.99). Missing of Tnc resulted in an equal Olig2 protein level in both immunized groups (*p* = 0.76).

**Figure 7:**
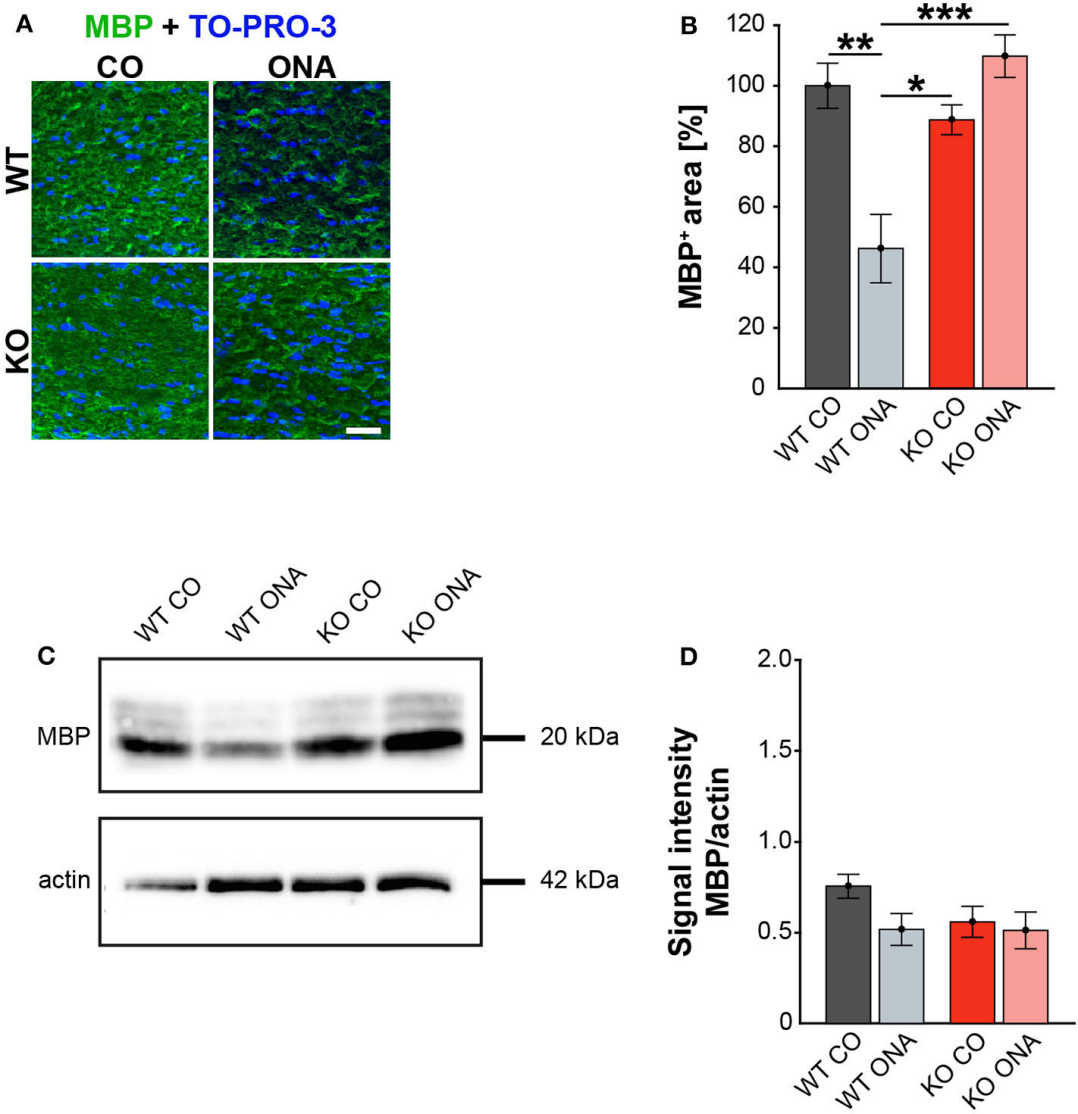
Unaltered MBP immunoreactivity post immunization in KO mice. **(A)** MBP (green) was stained in optic nerve tissue. In blue TO-PRO-3 detected cell nuclei. Immunohistochemistry indicates a reduced MBP signal in WT ONA. **(B)** A significant downregulation of MBP was noted in WT ONA compared to WT CO. Furthermore, the MBP signal was significantly reduced in WT ONA compared to control as well as to immunized KO mice. **(C)** Western blot analyses of MBP of optic nerve tissue. **(D)** Comparable MBP protein levels were observed in all groups. Data were analyzed using two-way ANOVA followed by Tukey's *post-hoc* test and values were indicated as mean ± SEM. *p < 0.05; **p < 0.01; ***p < 0.001. *n* = 5/group. Scale bar = 20 μm.

Finally, we also investigated MBP on protein level via immunohistochemistry and Western blot. This protein is specifically expressed by myelinating oligodendrocytes (61). Immunohistochemical staining revealed significantly reduced MBP immunoreactivity in WT ONA (Figure 7A). Statistical analyses discovered a decreased MBP signal in WT ONA (46.22 ± 11.30% MBP^+^ area) compared to WT CO (100.00 ± 7.47% MBP^+^ area, *p* = 0.001), KO CO (88.76 ± 4.93% MBP^+^ area, *p* = 0.01) and KO ONA (109.79 ± 7.01% MBP^+^ area *p* < 0.001, Figure 7B).

MBP was examined on protein level via Western blot analyses and a prominent protein band was detected at 20 kDa (Figure 7C). Quantitative analyses revealed comparable MBP protein levels in control (WT CO: 0.76 ± 0.07 a.u., KO CO: 0.56 ± 0.09 a.u.) and ONA mice (WT ONA: 0.52 ± 0.09 a.u., KO ONA: 0.51 ± 0.1 a.u., Figure 7D).

In conclusion, we found a significant decrease in mature as well as immature oligodendroglia in WT after immunization. Remarkably, immunized Tnc deficient mice showed no demyelination.

### Decreased Number of Microglia and Declined Microglial Response in KO ONA Mice

**Figure 8:**
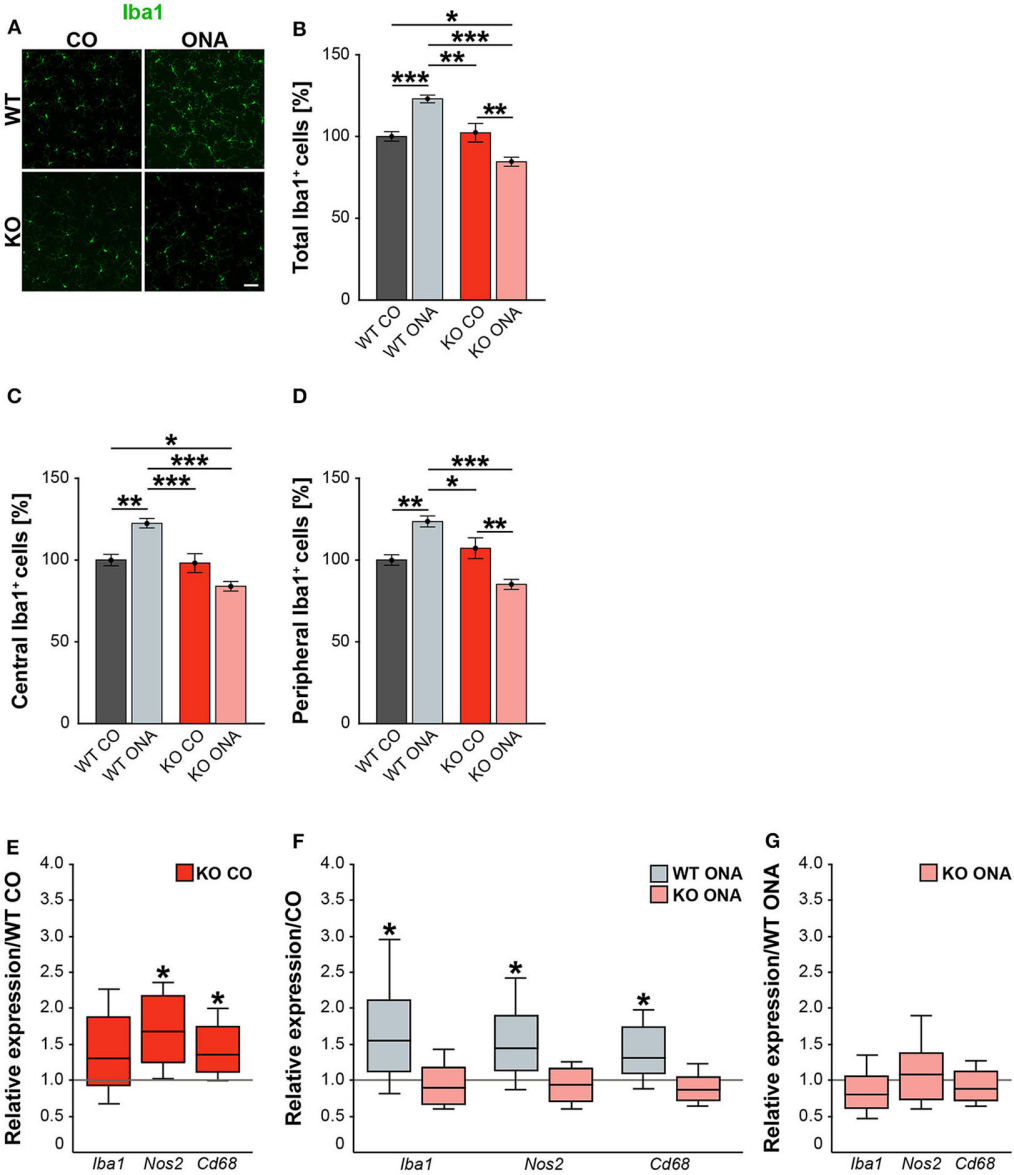
Decreased microglia response after immunization in KO mice. **(A)** Representative pictures of Iba1^+^ cells (green) in retinal flat-mounts of immunized and non-immunized WT and KO mice. **(B–D)** Quantification of Iba1^+^ microglia in control and immunized WT and KO animals in the total, central and peripheral retina (*n* = 9/group). WT ONA group exhibited clearly more microglia. In contrast, KO ONA animals displayed fewer Iba1^+^ cells. **(E)** RT-qPCR analyses (*n* = 5/group) of the relative *Iba1, Nos2*, and *Cd68* mRNA expression showed a significant increase of *Nos2* and *Cd68* in KO CO compared to WT CO retinae. No differences were observed for *Iba1* mRNA expression. **(F)** Compared to WT CO, a significant upregulation of *Iba1, Nos2*, and *Cd68* levels were found in WT ONA. No significant changes were detected regarding the expression levels of these markers in KO ONA compared to KO CO. **(G)** After immunization, a comparable mRNA level of *Iba1, Cd68*, and *Nos2* was detected in KO ONA compared to WT ONA. Data were analyzed using two-way ANOVA followed by Tukey's *post-hoc* test and present as mean ± SEM in **(B–D)**. For RT-qPCR, groups were compared using the pairwise fixed reallocation and randomization test and were shown as median ± quartile ± minimum/maximum in **(E–G)**. *p < 0.05; **p < 0.01; ***p < 0.001. Scale bar = 50 μm.

Neurodegeneration is often accompanied by reactive microgliosis. In order to analyze the microglia population in the EAG mouse model and the effects of immunization, we performed immunohistochemical staining of retinal flat-mounts using an Iba1 antibody (Figure 8A, Supplementary Table 4). The number of Iba1^+^ cells in the total as well as in the central and peripheral area of the retina was evaluated (Figures 8B–D). A significant increase in microglia numbers was detected in WT ONA (123.0 ± 2.4% Iba1^+^ cells) compared to control WT and KO in the total retina (WT CO: 100.0 ± 2.9% Iba1^+^ cells, *p* < 0.001 and vs. KO CO: 102.3 ± 5.7% Iba1^+^ cells; *p* = 0.002, Figure 8B). No differences were found between both control groups (*p* = 0.97). Remarkably, a significantly lower number of Iba1^+^ cells was observed after immunization in KO ONA (KO ONA: 84.5 ± 2.7% Iba1^+^ cells) compared to WT ONA (*p* < 0.001), WT CO (*p* = 0.03), and KO CO (*p* < 0.01). Also, in the central part of the retina 20% more Iba1^+^ cells were detected in WT ONA (122.5 ± 2.9% Iba1^+^ cells) compared to the corresponding control group (WT CO: 100.0 ± 3.5% Iba1^+^ cells, *p* < 0.01, Figure 8C). Immunized WT also showed significantly more microglial cells compared to control (*p* < 0.001) and immunized KO mice (*p* < 0.001). In KO CO, we counted 98.1 ± 5.8% Iba1^+^ cells, whereas KO ONA only has 84.0 ± 2.9% Iba1^+^ cells (*p* = 0.08). A reduced microglia number was noted in KO ONA compared to WT CO (*p* = 0.04). Equal numbers of microglial cells were seen in both control groups (*p* = 0.99). Similarly, the number of microglia in WT ONA (123.6 ± 3.4% Iba1^+^ cells) was significantly enhanced compared to WT CO (100.0 ± 3.2% Iba1^+^ cells, *p* = 0.002, Figure 8D) and KO CO (107.2 ± 6.4% Iba1^+^ cells, *p* < 0.05) in peripheral regions of the retinal. Also, the number of Iba1^+^ cells was lower in KO ONA (85.1 ± 3.1% Iba1^+^ cells) compared to KO CO (*p* < 0.01) and WT CO (*p* = 0.08) in the periphery. Additionally, a significantly reduced microglial response was detected in immunized KO and WT animals (*p* < 0.001). Regarding the quantification of Iba1^+^ cells in the periphery, both control groups had similar cell counts (*p* = 0.63).

In the next step, RT-qPCR was used to investigate whether microglia have reactive phenotypes. Besides *Iba1*, we also examined the markers *Nos2* and *Cd68* in retinal tissue (Figures 8E–G, Supplementary Table 5). No differences could be detected in the *Iba1* expression between control WT and KO mice (1.3-fold, *p* = 0.2, Figure 8E). However, KO CO mice showed significantly elevated levels of *Nos2* (1.7-fold; *p* = 0.013) and *Cd68* (1.3-fold; *p* = 0.017) compared to WT CO. The comparison of immunized and non-immunized WT illustrated a significantly increased expression of *Iba1* (1.5-fold, *p* = 0.048) as well as of the reactive markers *Nos2* (1.4-fold, *p* = 0.021) and *Cd68* (1.3-fold, *p* = 0.032, Figure 8F). Interestingly, comparable expression levels of these microglial markers were found in immunized KO and KO CO (*p* > 0.05, Figure 8F). RT-qPCR analyses revealed comparable mRNA levels in KO ONA compared to WT ONA (*p* > 0.05, Figure 8G).

In line with the RT-qPCR results of retinal tissue, we found a similar expression pattern of microglial markers in the optic nerve of control and immunized WT and KO mice (Supplementary Table 5, Supplementary Figure 2).

In summary, WT ONA animals showed a significantly increased microglia infiltration and glial marker expression, indicating an increased microglial response. Remarkably, a significantly reduced invasion and reactivity of microglia were observed in KO ONA, suggesting that Tnc signaling is an important modulator of microglia in glaucomatous neurodegeneration.

### Altered Expression Pattern of Pro- and Anti-inflammatory Cytokines in Immunized WT Compared to Immunized KO Animals

In our study, we noted a reactive gliosis and an increased microglial response after immunization in WT mice. Interestingly, these effects could not be detected in Tnc deficient animals.

**Figure 9:**
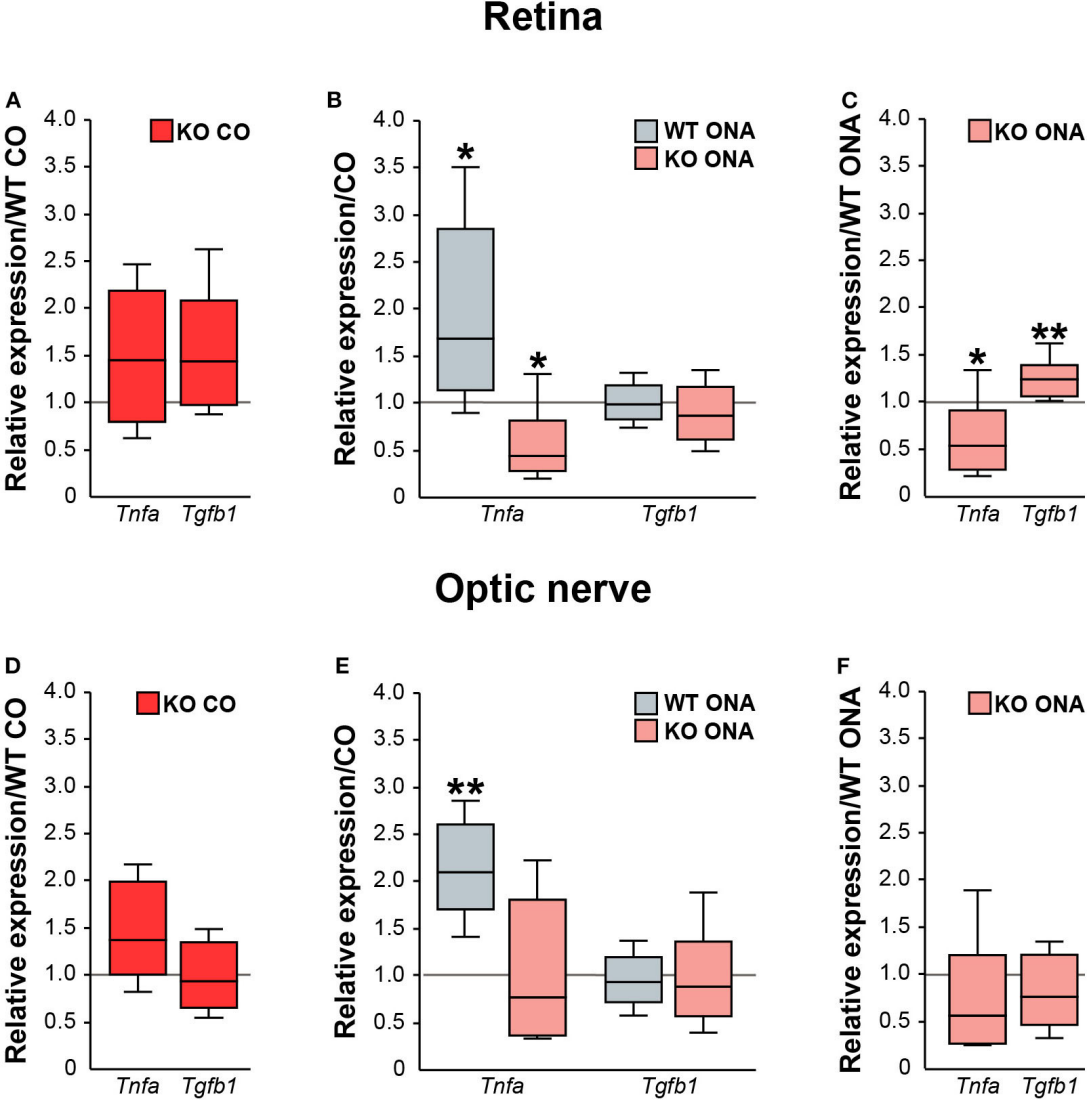
Reduced pro-inflammatory and enhanced anti-inflammatory cytokine expression in immunized KO mice. Relative expression of pro-inflammatory *Tnfa* and anti-inflammatory *Tgfb1* was examined via RT-qPCR in control and immunized WT and KO retinae **(A–C)** and optic nerves **(D–F)**. **(A)** Analysis revealed comparable levels of *Tnfa* and *Tgfb1* in KO CO compared to WT CO. **(B)** *Tnfa* expression level was significantly increased in WT ONA compared to WT CO. Strikingly, a reduced *Tnfa* mRNA level was found in KO ONA compared to the corresponding control group. Regarding *Tgfb1*, the expression was comparable in both genotypes. **(C)** Comparison of WT ONA and KO ONA. Here, the pro-inflammatory factor was downregulated and the anti-inflammatory cytokine was significantly upregulated in KO mice after immunization. **(D–F)** Quite similar expression patterns of both examined cytokines were noted in optic nerve tissue. Only, a significantly enhanced *Tnfa* expression was detected in WT ONA compared to WT CO **(E)**. Groups were compared using the pairwise fixed reallocation and randomization test and values were shown as median ± quartile ± maximum/minimum. *p < 0.05; **p < 0.01. *n* = 5/group.

Next, we analyzed the pro- and anti-inflammatory responses of the microglial phenotypes in retinae and optic nerves (Figure 9, Supplementary Table 5). Here, *Tnfa* was used to study M1 pro-inflammatory microglia, while *Tgfb1* is expressed by M2 anti-inflammatory microglia. RT-qPCR experiments revealed comparable mRNA level of *Tnfa* (1.4-fold, *p* = 0.132) and *Tgfb1* (1.4-fold, *p* = 0.071) in KO CO mice compared to WT CO mice (Figure 9A). After ONA-immunization, *Tnfa* was significantly upregulated in WT and interestingly downregulated in KO compared to the corresponding control groups (WT CO vs. WT ONA: 1.7-fold, *p* = 0.026 and KO CO vs. KO ONA: 0.4-fold, *p* = 0.031, Figure 9B). Statistical comparable *Tgfb1* mRNA levels were found in WT CO and WT ONA (1.0-fold; *p* = 0.807) as well as in KO CO and KO ONA (0.9-fold, *p* = 0.415; Figure 9B). The evaluation of both immunized genotypes showed a significant reduction of *Tnfa* (0.5-fold, *p* = 0.036) and a significant increase of *Tgfb1* (1.2-fold, *p* = 0.005) after immunization in KO mice (Figure 9C).

Finally, we examined, which microglial subtypes are altered due to an increased microglial reactivity in the optic nerve tissue of immunized and non-immunized WT and KO mice (Figures 9D–F, Supplementary Table 5). Equal mRNA levels of *Tnfa* (1.4-fold, *p* = 0.07) and *Tgfb1* (0.9-fold, *p* = 0.659) were seen in WT and KO controls (Figure 9D). A significantly enhanced *Tnfa* expression (2.1-fold, *p* = 0.008) and an unchanged *Tgfb1* expression (0.9-fold, *p* = 0.71) were detected in WT ONA compared to WT CO (Figure 9E). No changes of these markers were found in KO CO in comparison to KO ONA mice (*Tnfa*: 0.8-fold, *p* = 0.443 and *Tgfb1*: 0.9-fold, *p* = 0.575). In line with the RT-qPCR results of retinal tissue, we found a slightly reduced *Tnfa* (0.6-fold, *p* = 0.101) and an unaltered *Tgfb1* (0.8-fold, *p* = 0.297) expression between both immunized groups (Figure 9F).

In conclusion, missing Tnc resulted in a reduced mRNA level of pro-inflammatory *Tnfa*, but an enhanced expression of anti-inflammatory *Tgfb1*. The increased expression of the pro-inflammatory cytokine in WT after immunization points toward an enhanced presence of reactive M1 microglia.

## Discussion

Glaucoma involves a progressive degeneration of RGCs and their axons leading to visual field loss (62–64). Developing glaucoma is often associated with elevated IOP, but RGC damage can also occur without IOP changes. Previous studies provided evidence that an altered immune response is involved in glaucoma pathology (2, 65–68). In addition, a remodeling of ECM constituents was found in several retinal neurodegenerative diseases, including glaucoma (31–33, 69, 70).

In glaucoma pathology the mechanisms are currently poorly understood, especially the relationship between RGC loss and the role of the immune system as well as ECM molecules. Hence, we characterized glaucomatous damage associated with the absence of the ECM glycoprotein Tnc in an IOP-independent EAG mouse model for the first time. Therefore, we immunized WT and KO mice with ONA to induce retinal damage and analyzed IOP, retinal functionality, RGC degeneration, glial activation, and pro- and anti-inflammatory cytokine expression.

In a previous study, we already successfully transferred the EAG glaucoma model from rats to mice (49). Here, glaucomatous neurodegeneration was observed 6 weeks post ONA-immunization. However, in order to provoke a possible more robust glaucomatous macro- and microgliosis in our transgenic Tnc EAG mouse model, the duration of our study was extended to 10 weeks post immunization. Accordingly, we observed significantly increased GFAP levels and a higher number of Iba1^+^ infiltrating microglia cells in the WT, suggesting an induction of glaucomatous gliosis.

Our analyses revealed that the IOP of WT and KO stayed in normal ranges. Previous studies of the EAG animal model also showed no alteration in the IOP (49, 58). Comparable IOP in control and immunized animals points to the fact that the EAG model can be considered a suitable model for normal tension glaucoma.

Analyses of retinal functionality via ERG recordings revealed no differences in a- and b-wave responses in control and immunized WT and KO mice, which indicates that photoreceptor cells as well as bipolar and Müller glia cells are not affected in our model.

We found a significant loss of Brn3a^+^ RGCs in both genotypes following immunization. Interestingly, immunized KO mice displayed ~15% more RGCs in retinal flat-mounts compared to immunized WT mice. Moreover, a comparable number of RGCs was found in retinal cross-sections of KO ONA animals. We also verified a severe optic nerve damage by diminished βIII-tubulin staining in immunized WT, while no alterations were found in the KO ONA mice. Previous studies of the EAG rat model showed that antibody deposits are accompanied by apoptotic RGC death (71). Furthermore, activation of the complement system via the lectin pathway seems to trigger retinal degeneration in this model (72). In our present study, we demonstrate that Tnc deficiency results in an extenuated loss of RGCs. Accordingly, we suggest that beside immunological alterations, Tnc-mediated signaling pathways are involved in glaucoma pathology.

In an EAG rat model a significant loss of RGCs could be detected 22 days after immunization (71). While an early upregulation of Tnc and its interaction partner RPTPβ/ζ/phosphacan was observed already at 7 days (6). Additionally, we noted an increased Tnc immunoreactivity in the retina 14 days post immunization. However, we did not reveal any differences regarding the Tnc protein level at later points in time, namely 28 days after immunization. Therefore, we assume that alterations of Tnc levels occurred at an early point in time, shortly after immunization and then returned to normal values. Due to the fact that glaucomatous degeneration in both rodent EAG models is temporally comparable, we also assume that Tnc induction takes place at similar points in time. However, the precise timeline of Tnc induction should be determined in a future follow-up project. Ten weeks after immunization, we observed Tnc immunoreactivity in horizontal and amacrine cells as well as in the outer and inner plexiform layers (27, 28). The quantification of the Tnc signal intensity revealed comparable protein levels in control and immunized WT mice. This is in line with results from the EAG rat model, were no differences in the Tnc protein levels were noted at later points in time (6).

In regard to the temporal induction, *Tnc* expression levels were enhanced 3 days post injury and returned to normal levels 7 and 14 days after the induction of brain laser lesion (73). In the CNS, *Tnc* is re-expressed under pathological conditions and represents an important component of the glial scar (11, 25, 36, 74–76). In Alzheimer's disease, Tnc immunoreactivity is highly associated with amyloid-β plaques (20). In multiple sclerosis, Tnc has an immunomodulatory function and loss of Tnc protects from experimental autoimmune encephalopathy (24). In regard to brain injury it was demonstrated that Tnc isoforms that contain the fibronectin type III domains B and D as well as the smallest splice variants, were specifically upregulated after cortical lesion (77). Future studies should focus on comprehensive analyses of specific Tnc isoforms in our glaucoma model. Due to these and our results, we conclude that Tnc induction occurred at early points in time and returned to normal levels at later points in time after immunization with ocular antigens. We speculate that upregulation of Tnc may serve as an early indicator for neurodegenerative processes before retinal damage is detectable. Finally, we assume that Tnc is a key mediator of inflammatory responses in our glaucoma mouse model.

Studies reported that Tnc is involved in the pathogenesis of CNS autoimmunity and astrocytes are a main Tnc source (24, 29, 30, 34–36). Astrocytes are the major glial cells in the optic nerve head and provide neurotrophic as well as mechanical support for RGC axons. Furthermore, astrocytes influence survival and functionality of neurons (78, 79). Neuroinflammatory processes, characterized by altered functional properties and distribution of glial cells, appear to have an obvious function in glaucomatous optic neuropathy (80–82). In addition, an enhanced astrocyte reactivity in response to inflammatory signals is directly regulated by the ECM (83). Furthermore, RGC degeneration is accompanied by reactive astrogliosis, which results in a higher GFAP expression (51, 58, 84–86). In our study, we observed an upregulated *Gfap* expression and an increased GFAP immunostaining in retinal and optic nerve tissue of immunized WT mice, whereas absence of Tnc led to a missing reactive gliosis. Therefore, severe glaucomatous damage induced by immunization seems to be triggered by Tnc expressing reactive astrocytes in the WT condition. We assume that the increase of astrocytic Tnc results in an enhanced glial cell proliferation, infiltration and cytokine release.

We noted a reduced population of mature oligodendrocytes and OPCs after immunization in WT mice. Also, a reduced MBP immunoreactivity demonstrated a demyelination of the optic nerve in the WT condition. Furthermore, no evidence of a decreased oligodendroglia density and demyelination was detected after ONA-immunization in KO animals. Interestingly, the fraction of mature Olig2/CC1 double immunopositive oligodendrocytes was significantly increased in the KO, in agreement with findings that Tnc inhibits the maturation of oligodendrocytes (17, 87). In addition, a strong expression of Tnc in the optic nerve head inhibits the migration of oligodendrocytes from the optic nerve into the retina (88). Based on our results and the mentioned studies, we suggest that Tnc has an impact on demyelination processes. Missing of Tnc leads to no alteration in oligodendroglia but has a protective effect on myelination of optic nerve fibers in our EAG mouse model.

Microglia are the main resident immune cells of the CNS and play a crucial role during retinal neurodegeneration (89). They change their morphology to a reactive ameboid cell type after injury and neuroinflammatory changes in the retina occur during glaucomatous damage (81). Microglial activation is a very early event in glaucoma, often before significant loss of RGCs takes place (47, 90, 91). Analyses of microglial cells showed that Tnc deficiency results in a diminished microglial response characterized by a reduced number and reactivity. Furthermore, we detected enhanced levels of the anti-inflammatory factor *Tgfb1* and a decreased expression of the pro-inflammatory *Tnfa*. This is consistent with the study by Piccinini, which demonstrated that the deletion of Tnc reduced TNF-α production (92). In the retina, TGF-β signaling has an important role in neuronal differentiation and survival (93). In microglia, TGF-β regulates homeostasis and lack of TGF-β signaling promotes retinal degeneration (94, 95). Additionally, TGF-β inhibits pro-inflammatory cytokines and regulates proliferation and activity of microglia (96). Our results indicate that the loss of Tnc signaling in microglia may promote the neuroprotective M2-subtype, resulting in a weaker RGC loss as well as axonal fiber damage in immunized KO animals.

The WT condition exhibited unaltered *Tgfb1* expression and it is known that Tnc is able to bind this growth factor (97). In a pilocarpine seizure model it was shown that TGF-β signaling is accompanied by Tnc upregulation (98). Here, an increase of the *Tgfb1* level occurred shortly after pilocarpine application, whereas the rise of *Tgfb*1 was less pronounced over time. Based on this date, we suggest that TGF-β dependent signaling in WT mice is associated with an early upregulation of Tnc and becomes less efficient at later points in time.

An enhanced microglial infiltration and marker expression revealed an increased glial response in WT post immunization in our study. Moreover, we found a significantly increased expression of the pro-inflammatory cytokine *Tnfa*. In glaucoma, a glia-derived neuronal death was described through TNF-α (99–102). The indirect neurotoxicity of this pro-inflammatory factor is based on TNF receptor 2-mediated activation of microglia, whereas blocking of TNF-α results in an extenuated microglial response (101). Moreover, TNF-α does not directly induce RGC death but leads to an upregulation of the microglial Fas ligand, where the membrane-bound form elicits RGC apoptosis (103, 104). However, in an early phase after injury a neuroprotective effect of TNF-α was found in an optic nerve crush animal model (105).

Microglia are involved in time-dependent astrocytic reactivity and this cross-talk is mediated by cytokines such as TNF-α (106–108). Previous studies noted that resting microglia switched to a M1-like phenotype, which can lead to neurotoxic effects by producing high levels of pro-inflammatory cytokines (43, 109, 110). Based on this, we speculate that microglia are the first glial cells that react after immunization as a driving force of reactive gliosis in the EAG mouse model. Our investigations of immunized WT and Tnc deficient mice lead to the assumption that Tnc promotes immunomodulatory processes of the neurotoxic M1-subtype in turns of higher activity. This is in line with previous reports that Tnc supports the activity of M1-microglia (36, 111).

Astrocytes as well as microglia express the toll-like receptor 4 (TLR4) and its expression is maintained by the extracellular environment (112, 113). Tnc activates TLR4 via its fibrinogen-like globe domain and thereby regulates the production of pro-inflammatory cytokines, such as IL-6, IL-8, and TNF-α (114, 115). In primary microglia, Tnc induced the synthesis of *Tnfa* and regulated the expression of *iNOS* via TLR4 signaling (38). Besides microglia, activated macrophages can also produce TNF-α (116). In human macrophages, specific alternatively spliced Tnc isoforms stimulate IL-6 and TNF-α release via TLR4 activation (117). In an experimental model of retinal degeneration, infiltration of peripheral monocytes occurred very early on, before microglia activation could be observed (118). Accordingly, we conclude that enhanced *Tnfa* expression, 10 weeks after immunization, is caused by Tnc-induced microglial TLR4 activation. Furthermore, we speculate that microglial TNF-α release and Tnc itself may regulate astrocytic *Tnc* expression, which results in a subsequently harmful astrogliosis in glaucomatous WT mice.

Our study could show that Tnc signaling pathways modulate both microglia and astrocyte response during glaucomatous damage. This might lead to a feedback loop by which increased levels of M1-microglial factors impact astrocytic Tnc release and vice versa. Finally, we propose that Tnc acts as an endogenous TLR4 ligand and represents an alarmin in our glaucoma model. However, it is possible that other receptors, for instance integrins, have an impact on inflammatory effects of Tnc (119, 120). In this regard, also other ECM components and Tnc interaction partners might be involved. Here, e.g., the extra domain A of fibronectin was recently described to elevate IOP through TLR4 signaling in a TGFβ2-induced ocular hypertension mouse model (121). Hence, future studies should focus on the downstream signaling cascade of TLR4 in our IOP-independent EAG mouse model.

## Conclusion

Taken together, our study demonstrated that Tnc influences glial response, migration, and inflammation during glaucomatous damage. This model is ideally suited for a better understanding of the molecular mechanisms between retinal neurodegeneration and ECM remodeling in order to develop future therapeutic options.

## Data Availability Statement

All datasets generated for this study are included in the article/Supplementary Material.

## Ethics Statement

The animal study was reviewed and approved by Landesamt für Natur, Umwelt und Verbraucherschutz, North Rhine-Westphalia.

## Author Contributions

SW performed experiments, analyzed data, and wrote the manuscript. JR designed the study, analyzed data, and revised the manuscript. SR and ZC performed experiments and analyzed data. SCJ and AF designed the study and revised the manuscript. All authors read and approved the final manuscript.

## Conflict of Interest

The authors declare that the research was conducted in the absence of any commercial or financial relationships that could be construed as a potential conflict of interest.

## Abbreviations

Brn3a: brain-specific homeobox/POU domain protein 3a
CC1: coiled coil-1
Cd68: cluster of differentiation 68
EAG: experimental autoimmune glaucoma
ECM: extracellular matrix
ERG: electroretinogram
GCL: ganglion cell layer
GFAP: glial fibrillary acidic protein
Iba1: ionized calcium-binding adapter molecule 1
INL: inner nuclear layer
iNOS: inducible nitric oxide synthase
IOP: intraocular pressure
IPL: inner plexiform layer
KO: knockout
KO CO: control group tenascin-C knockout
KO ONA: immunized tenascin-C knockout
MBP: myelin basic protein
NFL: nerve fiber layer
Nos2: nitric oxide synthase 2
Olig2: oligodendrocyte transcription factor 2
ONA: optic nerve antigen
ONL: outer nuclear layer
OPC: oligodendrocytes precursor cell
OPL: outer plexiform layer
RGC: retinal ganglion cell
*Tgfb*/TGF-β: transforming growth factor-beta
Tnc: tenascin-C
TLR4: toll-like receptor 4
*Tnfa*/TNF-α: tumor necrosis factor-alpha
WT: wild type
WT CO: control group wild type
WT ONA: immunized wild type.
