# Supplementary material for: Loss of the Extracellular Matrix Molecule Tenascin-C Leads to Absence of Reactive Gliosis and Promotes Anti-inflammatory Cytokine Expression in an Autoimmune Glaucoma Mouse Model
Source: Front Immunol. 2020 Oct 9;11:566279. doi: 10.3389/fimmu.2020.566279 (PMC7581917; doi:10.3389/fimmu.2020.566279)
Supplement: Supplementary file 1 [file Table_1.pdf]

Supplementary Material

1 Supplementary Tables

Supplementary Table 1:
IOP measurements before and after immunization in WT and KO mice.

Genotype	Group	Age (weeks)	Mean	SEM	P-value	N
WT	-	5	9.8	0.2	1.0	16
KO	-	5	9.7	0.1		
WT	CO	6	9.4	0.2	>0.05	8
WT	ONA	6	9.2	0.3		
KO	CO	6	9.1	0.3		
KO	ONA	6	10.3	0.3		
WT	CO	7	9.8	0.4	>0.05	8
WT	ONA	7	9.9	0.4		
KO	CO	7	9.9	0.3		
KO	ONA	7	9.9	0.2		
WT	CO	8	11.2	0.5	>0.05	8
WT	ONA	8	11.3	0.3		
KO	CO	8	10.1	0.3		
KO	ONA	8	10.2	0.4		
WT	CO	9	10.2	0.3	>0.05	8
WT	ONA	9	10.9	0.4		
KO	CO	9	10.8	0.3		
KO	ONA	9	10.2	0.6		
WT	CO	10	10.1	0.4	>0.05	8
WT	ONA	10	9.7	0.3		
KO	CO	10	10.6	0.4		
KO	ONA	10	10.5	0.4		
WT	CO	11	11.0	0.3	>0.05	8
WT	ONA	11	10.6	0.2		
KO	CO	11	10.9	0.7		
KO	ONA	11	10.6	0.3		
WT	CO	12	10.8	0.2	>0.05	8
WT	ONA	12	10.8	0.3		
KO	CO	12	10.3	0.4		
KO	ONA	12	10.2	0.3		
WT	CO	13	10.2	0.3	>0.05	8
WT	ONA	13	9.5	0.3		
KO	CO	13	10.5	0.4		
KO	ONA	13	10.3	0.4		

WT	CO	14	9.4	0.1	>0.05	8
WT	ONA	14	9.4	0.2		
KO	CO	14	9.3	0.3		
KO	ONA	14	10.8	0.4		
WT	CO	15	9.7	0.2	>0.05	8
WT	ONA	15	10.3	0.2		
KO	CO	15	10.1	0.5		
KO	ONA	15	10.2	0.5		

Supplementary Table 2:
Data of a- and b-wave amplitudes recorded from WT CO, WT ONA, KO CO, and KO ONA animals. Values of light flash intensity (cd x s/m²) are displayed as mean ± SEM (n = 5/group).

**Light flash intensity [cd x s/m²]**	**0.1**		**0.3**		**1**		**3**		**10**		**25**	
**Amplitude [µV]**	**Mean**	**SEM**	**Mean**	**SEM**	**Mean**	**SEM**	**Mean**	**SEM**	**Mean**	**SEM**	**Mean**	**SEM**
**A-wave**												
WT CO	40.1	9.9	69.4	14.5	99.71	13.9	112.3	20.6	129.6	26.9	148.0	6.3
WT ONA	39.4	4.1	60.1	4.9	91.02	3.8	90.8	5.8	108.1	9.1	106.6	8.2
KO CO	27.4	3.2	41.4	7.2	68.87	13.9	87.6	13.6	114.5	20.1	103.8	26.5
KO ONA	35.8	2.6	67.8	7.2	80.80	4.7	92.3	3.3	109.9	14.8	151.6	16.2
P-value	> 0.05		> 0.05		> 0.05		> 0.05		> 0.05		> 0.05	
**B-wave**												
WT CO	279.4	32.9	335.7	43.3	333.98	41.5	302.2	41.9	355.1	48.2	354.8	37.9
WT ONA	250.6	21.9	331.3	27.5	348.31	32.4	365.5	41.1	356.4	35.7	381.9	48.9
KO CO	169.0	25.9	188.9	39.9	228.82	39.1	247.0	38.7	249.3	49.2	330.9	38.8
KO ONA	233.1	18.4	284.0	28.8	309.79	33.0	307.6	24.6	307.8	30.6	318.0	34.7
P-value	> 0.05		> 0.05		> 0.05		> 0.05		> 0.05		> 0.05	

Supplementary Table 3:

Analyses of Tnc protein levels via immunohistochemistry and Western blot in control and immunized WT retinae.

**Geno-type**	**Group**	**Method**	**Mean**	**SEM**	**P-value**	**N**
**Tnc^{+} area [%]**						
WT	CO	IHC	100.0	18.6	0.40	4
WT	ONA		130.3	28.0		
**a.u.**						
WT	CO	Western blot	0.66	0.09	0.45	5
WT	ONA		0.56	0.10		

Supplementary Table 4:
Cell counts of Brn3a^{+} and Iba1^{+} cells [%] in WT CO, WT ONA, KO CO, and KO ONA animals. WT CO group was set to 100%. P-values < 0.05 are shown in bold.

Geno-type	Group	Retinal area	Tissue	Mean	SEM	P-value	N
**Brn3a^{+} cells [%]**							
WT	CO	-	Cross-section	100.0	4.2	**0.004**$^{\textbf{WT CO vs. WT ONA}}$<br>0.64$^{\text{WT CO vs. KO CO}}$<br>0.10$^{\text{WT CO vs. KO ONA}}$<br>**0.04**$^{\textbf{WT ONA vs. KO CO}}$<br>0.39$^{\text{WT ONA vs. KO ONA}}$<br>0.57$^{\text{KO CO vs. KO ONA}}$	5
WT	ONA			73.1	6.1		
KO	CO			92.2	3.9		
KO	ONA			83.7	8.7		
WT	CO	Central	Flat-mount	100.0	2.5	**< 0.001**$^{\textbf{WT CO vs. WT ONA}}$<br>0.94$^{\text{WT CO vs. KO CO}}$<br>**0.007**$^{\textbf{WT CO vs. KO ONA}}$	9
		Peripheral		100.0	1.7	**< 0.001**$^{\textbf{WT CO vs. WT ONA}}$<br>0.99$^{\text{WT CO vs. KO CO}}$<br>**0.01**$^{\textbf{WT CO vs. KO ONA}}$	
		Total		100.0	2.0	**< 0.001**$^{\textbf{WT CO vs. WT ONA}}$<br>0.96$^{\text{WT CO vs. KO CO}}$<br>**0.01**$^{\textbf{WT CO vs. KO ONA}}$	
WT	ONA	Central		82.7	1.7	**< 0.001**$^{\textbf{WT ONA vs. WT CO}}$<br>**0.003**$^{\textbf{WT ONA vs. KO CO}}$<br>0.80$^{\text{WT ONA vs. KO ONA}}$	9
		Peripheral		77.0	1.8	**< 0.001**$^{\textbf{WT ONA vs. WT CO}}$<br>**< 0.001**$^{\textbf{WT ONA vs. KO CO}}$<br>0.06$^{\text{WT ONA vs. KO ONA}}$	
		Total		80.3	1.5	**< 0.001**$^{\textbf{WT ONA vs. WT CO}}$<br>**< 0.001**$^{\textbf{WT ONA vs. KO CO}}$<br>0.27$^{\text{WT ONA vs. KO ONA}}$	
KO	CO	Central		97.8	2.9	0.94$^{\text{KO CO vs. WT CO}}$<br>**0.003**$^{\textbf{KO CO vs. WT ONA}}$<br>**0.03**$^{\textbf{KO CO vs. KO ONA}}$	9
		Peripheral		99.0	4.1	0.99$^{\text{KO CO vs. WT CO}}$<br>**< 0.001**$^{\textbf{KO CO vs. WT ONA}}$<br>**0.02**$^{\textbf{KO CO vs. KO ONA}}$	
		Total		98.2	3.3	0.96$^{\text{KO CO vs. WT CO}}$<br>**< 0.001**$^{\textbf{KO CO vs. WT ONA}}$<br>**0.02**$^{\textbf{KO CO vs. KO ONA}}$	
KO	ONA	Central		86.3	3.7	**0.007**$^{\textbf{KO ONA vs. WT CO}}$<br>0.80$^{\text{KO ONA vs. WT ONA}}$<br>**0.03**$^{\textbf{KO ONA vs. KO CO}}$	9
		Peripheral		87.1	2.8	**0.01**$^{\textbf{KO ONA vs. WT CO}}$<br>0.06$^{\text{KO ONA vs. WT ONA}}$<br>**0.02**$^{\textbf{KO ONA vs. KO CO}}$	
		Total		86.9	3.1	**0.01**$^{\textbf{KO ONA vs. WT CO}}$	

						0.27[KO ONA vs. WT ONA]<br>**0.02[KO ONA vs. KO CO]**	
**Iba1⁺ cells [%]**							
WT	CO	Central	Flat-mount	100.0	3.5	**0.002[WT CO vs. WT ONA]**<br>0.99[WT CO vs. KO CO]<br>**0.04[WT CO vs. KO ONA]**	9
		Peripheral		100.0	3.2	**0.002[WT CO vs. WT ONA]**<br>0.62[WT CO vs. KO CO]<br>0.08[WT CO vs. KO ONA]	
		Total		100.0	2.9	**< 0.001[WT CO vs. WT ONA]**<br>0.97[WT CO vs. KO CO]<br>**0.03[WT CO vs. KO ONA]**	
WT	ONA	Central		122.5	2.9	**0.002 [WT ONA vs. WT CO]**<br>**< 0.001[WT ONA vs. KO CO]**<br>**< 0.001[WT ONA vs. KO ONA]**	9
		Peripheral		123.6	3.4	**0.002 [WT ONA vs. WT CO]**<br>**< 0.05[WT ONA vs. KO CO]**<br>**< 0.001[WT ONA vs. KO ONA]**	
		Total		123.0	2.4	**< 0.001[WT ONA vs. WT CO]**<br>**0.002[WT ONA vs. KO CO]**<br>**< 0.001[WT ONA vs. KO ONA]**	
KO	CO	Central		98.1	5.8	0.08[KO CO vs. KO ONA]<br>0.99[KO CO vs. WT CO]<br>**< 0.001[KO CO vs. WT ONA]**	9
		Peripheral		107.2	6.4	**0.004[KO CO vs. KO ONA]**<br>0.63[KO CO vs. WT CO]<br>**< 0.05[KO CO vs. WT ONA]**	
		Total		102.3	5.7	**0.009[KO CO vs. KO ONA]**<br>0.97[KO CO vs. WT CO]<br>**0.002[KO CO vs. WT ONA]**	
KO	ONA	Central		84.0	2.9	0.08[KO ONA vs. KO CO]<br>**0.04[KO ONA vs. WT CO]**<br>**< 0.001[KO ONA vs. WT ONA]**	9
		Peripheral		85.1	3.1	**0.004[KO ONA vs. KO CO]**<br>0.08[KO ONA vs. WT CO]<br>**< 0.001[KO ONA vs. WT ONA]**	
		Total		84.5	2.7	**0.009[KO ONA vs. KO CO]**<br>**0.03[KO ONA vs. WT CO]**<br>**< 0.001[KO ONA vs. WT ONA]**	

Supplementary Table 5:
RT-qPCR analyses of glial cell types and pro- and anti-inflammatory cytokines in WT CO, WT ONA, KO CO, and KO ONA animals, the fold change of the expression is displayed. P-values < 0.05 are shown in bold (n = 5/group).

Genotype/ group	Gene	Tissue	Median	Quartile + maximum/minimum	P-value
WT CO vs. KO CO	*Gfap*	Retina	1.4	1.012 - 2.016<br>0.724 - 2.500	0.110
		Optic nerve	1.1	0.799 - 1.406<br>0.645 - 1.531	0.539
WT CO vs. WT ONA		Retina	1.7	1.011 - 2.751<br>0.902 - 3.380	**0.044**
		Optic nerve	1.4	0.970 - 1.923<br>0.845 - 2.083	0.071
KO CO vs. KO ONA		Retina	1.2	0.812 - 1.817<br>0.614 - 2.557	0.362
		Optic nerve	0.5	0.300 - 0.918<br>0.174 - 1.491	**0.047**
WT ONA vs. KO ONA		Retina	1	0.625 - 1.518<br>0.450 - 2.136	0.993
		Optic nerve	0.4	0.242 - 0.705<br>0.128 - 1.150	**0.021**
WT CO vs. KO CO	*Iba1*	Retina	1.3	0.924 - 1.869<br>0.665 - 2.254	0.201
		Optic nerve	1.1	0.757 - 1.502<br>0.614 - 1.827	0.607
WT CO vs. WT ONA		Retina	1.5	1.124 - 2.100<br>0.816 - 2.941	**0.048**
		Optic nerve	1.5	1.117 - 2.068<br>0.860 - 2.515	**0.032**
KO CO vs. KO ONA		Retina	0.9	0.672 - 1.182<br>0.611 - 1.424	0.399
		Optic nerve	0.9	0.584 - 1.526<br>0.437 - 2.189	0.842
WT ONA vs. KO ONA		Retina	0.8	0.629 - 1.063<br>0.478 - 1.363	0.2
		Optic nerve	0.7	0.463 - 1.160<br>0.327 - 1.436	0.148
WT CO vs. KO CO	*Nos2*	Retina	1.7	1.240 - 2.151<br>1.006 - 2.342	**0.013**
		Optic nerve	1.2	0.881 - 1.618<br>0.648 - 2.009	0.292
WT CO vs. WT ONA		Retina	1.4	1.144 - 1.892<br>0.873 - 2.408	**0.021**
		Optic nerve	1.4	1.095 - 1.633	**0.008**

				0.975 - 1.888	
KO CO vs. KO ONA		Retina	0.9	0.716 - 1.172	0.605
				0.614 - 1.261	
		Optic nerve	0.6	0.372 - 1.224	0.197
				0.238 - 1.829	
WT ONA vs. KO ONA		Retina	1.1	0.743 - 1.381	0.65
				0.618 - 1.897	
		Optic nerve	0.6	0.298 - 1.093	**0.043**
				0.236 - 1.377	
WT CO vs. KO CO	*Cd68*	Retina	1.3	1.105 - 1.731	**0.017**
				0.986 - 1.985	
		Optic nerve	1.2	0.956 - 1.644	0.149
				0.758 - 2.009	
WT CO vs. WT ONA		Retina	1.3	1.101 - 1.732	**0.032**
				0.893 - 1.976	
		Optic nerve	1.1	0.678 - 1.926	0.752
				0.530 - 2.763	
KO CO vs. KO ONA		Retina	0.9	0.735 - 1.049	0.153
				0.654 - 1.226	
		Optic nerve	0.9	0.572 - 1.504	0.685
				0.444 - 2.403	
WT ONA vs. KO ONA		Retina	0.9	0.731 - 1.133	0.3
				0.646 - 1.283	
		Optic nerve	1.0	0.534 - 1.846	0.93
				0.323 - 3.403	
WT CO vs. KO CO	*Tnfa*	Retina	1.4	0.795 – 2.186	0.132
				0.612 – 2.466	
		Optic nerve	1.4	1.011 - 1.984	0.07
				0.818 - 2.177	
WT CO vs. WT ONA		Retina	1.7	1.131 - 2.836	**0.026**
				0.884 - 3.491	
		Optic nerve	2.1	1.712 - 2.615	**0.008**
				1.414 - 2.868	
KO CO vs. KO ONA		Retina	0.4	0.276 - 0.810	**0.031**
				0.187 - 1.305	
		Optic nerve	0.8	0.366 - 1.813	0.443
				0.335 - 2.231	
WT ONA vs. KO ONA		Retina	0.5	0.288 - 0.916	**0.036**
				0.210 - 1.335	
		Optic nerve	0.6	0.278 - 1.202	0.101
				0.255 - 1.902	
WT CO vs. KO CO	*Tgfb*	Retina	1.4	0.969 – 2.078	0.071
				0.866 - 2.625	
		Optic nerve	0.9	0.660 - 1.349	0.659
				0.554 - 1.491	
		Retina	1.0	0.820 - 1.184	0.807

WT CO vs. WT ONA				0.732 - 1.316	
		Optic nerve	0.9	0.716 - 1.204	0.710
				0.583 – 1.372	
KO CO vs. KO ONA		Retina	0.9	0.614 - 1.174	0.415
				0.490 - 1.351	
		Optic nerve	0.9	0.575 - 1.373	0.575
				0.403 - 1.878	
WT ONA vs. KO ONA		Retina	1.2	1.058 - 1.386	**0.005**
				1.008 - 1.612	
		Optic nerve	0.8	0.479 - 1.222	0.297
				0.333 - 1.785	

1.1 Supplementary Figures

Supplementary Figure 1: Unaltered Tnc protein levels 10 weeks post immunization in WT retinae. **(A)** Retinal cross-sections were stained with an antibody against Tnc (red) and TO-PRO-3 (blue) in control and immunized WT mice (n = 4/group). **(B)** Quantification of Tnc immunoreactivity showed no differences in WT CO and WT ONA group (p = 0.40). **(C)** Western blots revealed no changes in both groups (n = 5/group). **(D)** Quantification of signal intensity verified no alterations in control and immunized WT animals (p = 0.45). Data were analyzed via Student *t*-test and presented as mean ± SEM. Scale bar = 20 µm. ONL: outer nuclear layer, OPL: outer plexiform layer, INL: inner nuclear layer, IPL: inner plexiform layer, GCL: ganglion cell layer.

Supplementary Figure 2: RT-qPCR analyses of optic nerve tissue from control and immunized WT and KO mice.
(A) Examination of relative *Iba1*, *Nos2,* and *Cd68* mRNA expression showed no changes in KO CO compared to WT CO. **(B)** Compared to WT CO, a significant upregulation of *Iba1* and *Nos2* levels was verified in WT ONA. While no significant changes were detected regarding the expression levels of these markers in KO ONA compared to KO CO. **(C)** After immunization, a significantly downregulation of *Nos2* expression was observed in KO ONA compared to WT ONA, whereas comparable mRNA level of *Iba1 and Cd68* were detected in KO ONA vs. WT ONA. Groups were compared using the pairwise fixed reallocation and randomization test and were shown as median ± quartile ± minimum/maximum. n = 5/group. *p < 0.05; **p < 0.01.
