# Supplementary figures and images for: Loss of the Extracellular Matrix Molecule Tenascin-C Leads to Absence of Reactive Gliosis and Promotes Anti-inflammatory Cytokine Expression in an Autoimmune Glaucoma Mouse Model
Source: Front Immunol. 2020 Oct 9;11:566279. doi: 10.3389/fimmu.2020.566279 (PMC7581917; doi:10.3389/fimmu.2020.566279)

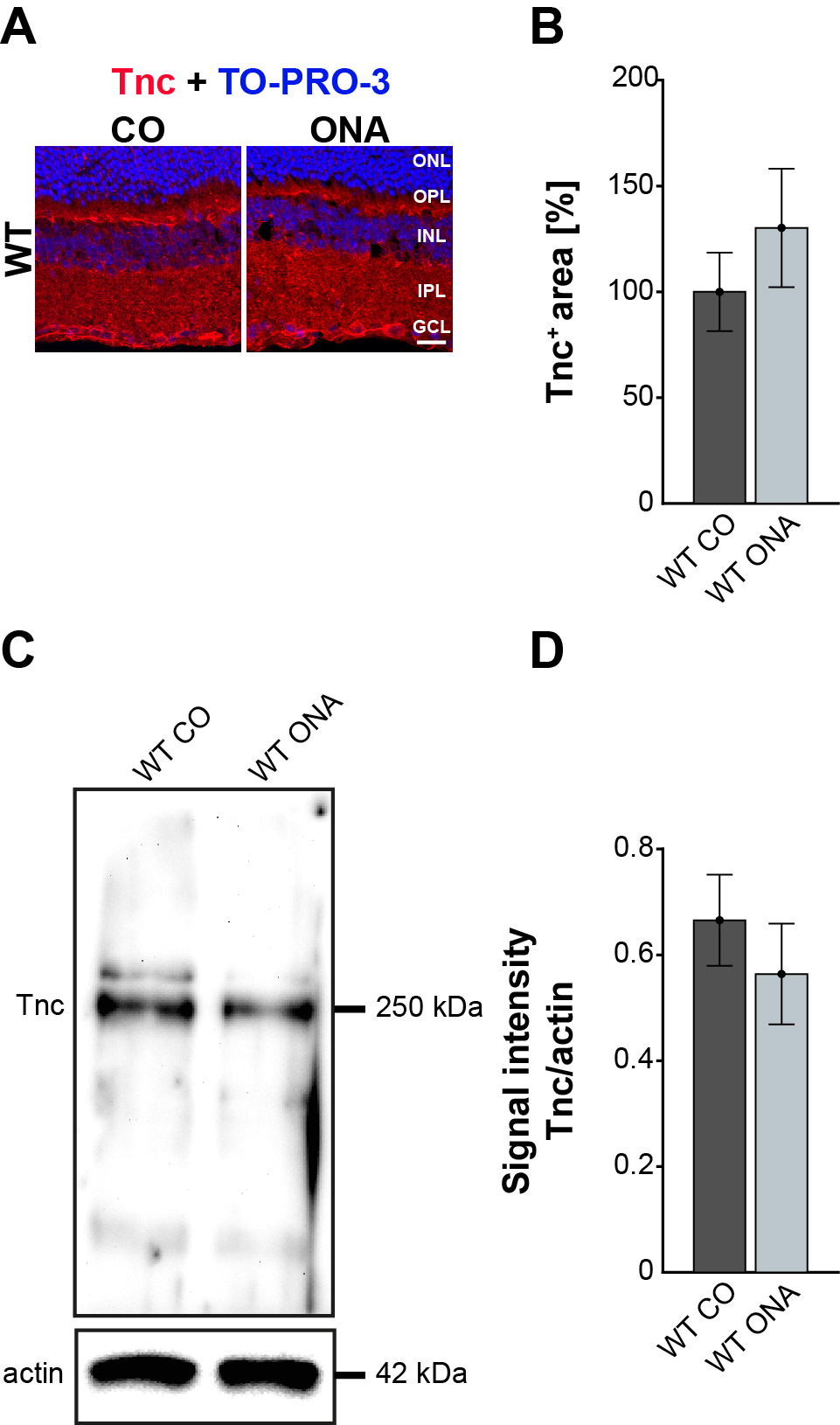

Supplement: Supplementary file 2 [file Image_1.jpg]

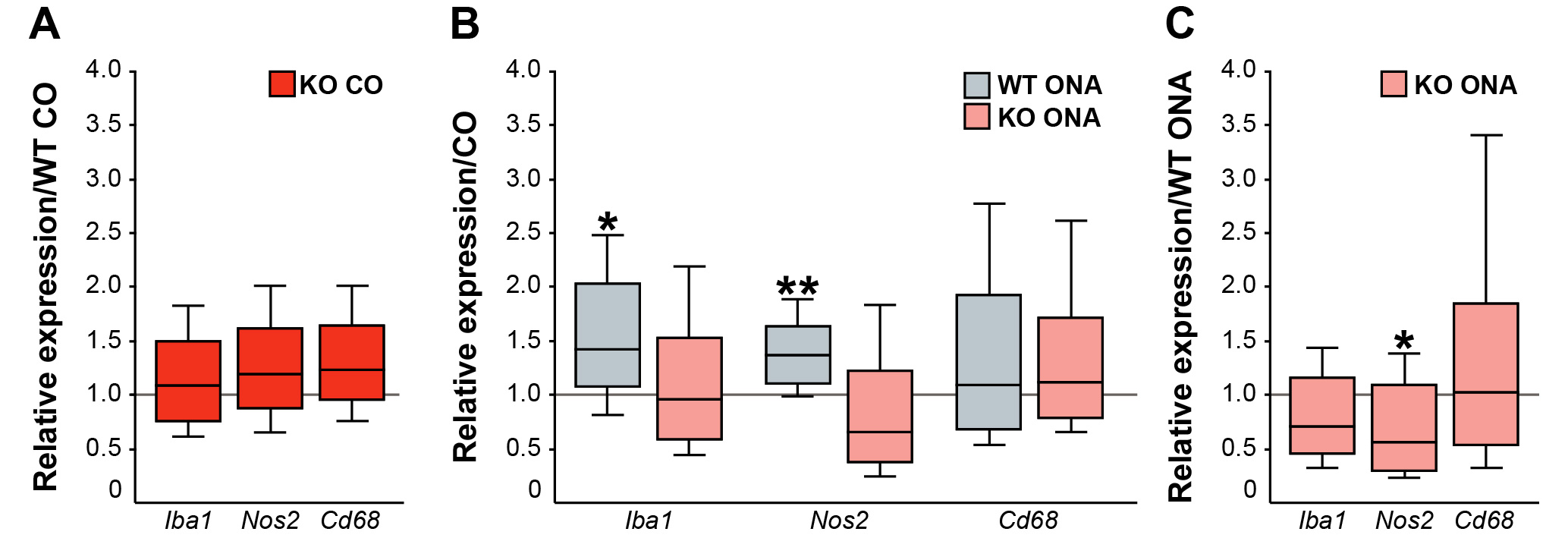

Supplement: Supplementary file 3 [file Image_2.jpg]
